# Projecting global oil palm expansion under zero-deforestation commitments: Direct and indirect land use change impacts

**DOI:** 10.1016/j.isci.2023.106971

**Published:** 2023-05-26

**Authors:** Floris Leijten, Uris Lantz C Baldos, Justin A. Johnson, Sarah Sim, Peter H. Verburg

**Affiliations:** 1 Environmental Geography Group, Institute for Environmental Studies (IVM), Vrije Universiteit Amsterdam, 1081HV Amsterdam, the Netherlands; 2 Department of Agricultural Economics, Purdue University, 403 West State Street, West Lafayette, IN 47907, USA; 3 Institute on the Environment, University of Minnesota, Saint Paul, MN 55108, USA; 4 Safety and Environmental Assurance Centre, Unilever R&D, Colworth Science Park, Sharnbrook, Bedfordshire, UK; 5 Swiss Federal Institute for Forest, Snow and Landscape Research, Birmensdorf, Switzerland

**Keywords:** Environmental management, Environmental policy, Environmental analysis, Agricultural science, Land use

## Abstract

In the last three decades, global production of oil palm has boomed, which has partly come at the expense of tropical rainforests. Recognizing this, many companies operating in the palm oil industry have committed to eliminate deforestation from their operations, often referred to as zero-deforestation commitments (ZDCs). Here, we estimate that if ZDCs are fully adopted and enforced across all sectors and geographies, the global extent of oil palm plantations may be 11 M ha or 40% smaller in 2030 than in a business-as-usual (BAU) scenario that assumes no compliance with ZDCs. As a result of such land-sparing effects, we estimate that 96 M ha of forests are saved from conversion, of which, 17% would otherwise have been converted (directly or indirectly) due to expanding oil palm plantations. Overall, these figures suggest that ZDCs have the potential to deliver major environmental benefits if they are fully adopted and enforced.

## Highlights

• Many companies have committed to reduce deforestation from their supply chains
• Over 16 M ha of forest could be cleared by oil palm plantations
• We estimate that zero-deforestation commitments could avoid 96 M ha of forest loss
• Sector-wide commitments thus hold potential for protecting forests across the globe

## Graphical abstract

**Figure:**
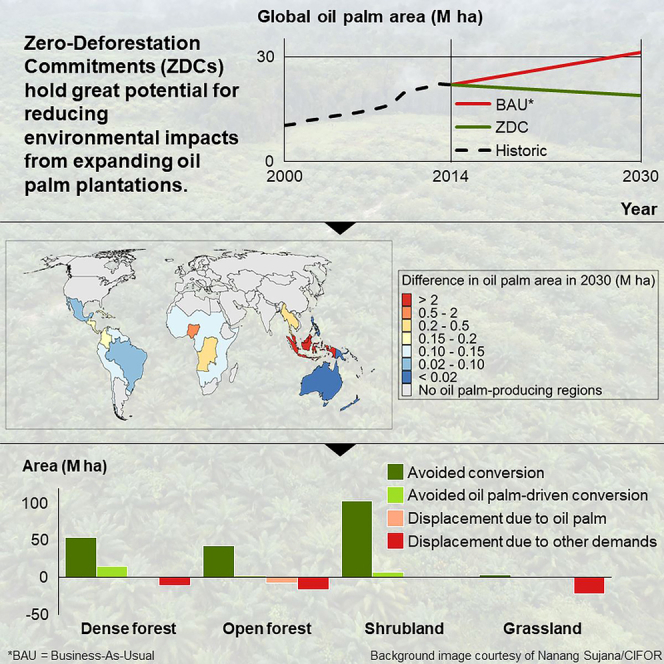

## Introduction

In the last three decades, global production of oil palm (*Elaeis guineensis*) fresh fruit bunches (FFBs) has boomed, increasing by nearly 600%.^1^ Oil palm provides two types of vegetable oil: palm oil and palm kernel oil. These oils are used in a variety of applications including foods, soaps, detergents, cosmetics, pharmaceuticals, and biofuels.^2^ Although the yields vary substantially depending on the age of the plantation,^3^ the time-averaged yields of palm oil per hectare are by far the highest of all oil crops.^4^ Over the last decades, palm oil has become the most consumed and traded vegetable oil in the world and demand will very likely continue to grow in the near future.^5^^,^^6^^,^^7^ However, in contrast to other major agricultural commodities such as rice or wheat, increased demand for oil palm has mostly been met by expanding the total area under production rather than intensification (i.e. increasing yields on existing production areas).^8^ This has resulted in an increased area of oil palm plantations of more than 350%—from 6 to 28 M ha—since 1990 (see Figure S1).^1^ As oil palm only grows in the tropics, this expansion has partly come at the expense of tropical rainforests.^9^ This is especially true for Southeast Asia, which is the largest palm oil-producing region of the world, accounting for 84% of production in 2018.^1^ It is estimated that around 45% of all new oil palm plantations established in Southeast Asia since 1989 have replaced forests.^10^ At the same time, there are large opportunities for the expansion of oil palm plantations outside tropical forests^11^^,^^12^ and recent evidence suggests that recent oil palm expansion in Latin America has been largely deforestation-free.^13^^,^^14^ In recent years, many companies involved in the palm oil industry have pledged to eliminate or reduce deforestation from their supply chains. These pledges are often referred to as zero-deforestation commitments (ZDCs).^15^ ZDCs apply to forests within a company’s supply chain, regardless of whether that company owns the land or not. As of April 2020, 83% of the palm oil refining capacity in Indonesia and Malaysia^1^—which, together, accounted for 84% of global production between 2010 and 2019—was covered by ZDCs.^16^^,^^17^ While various studies have considered the past effectiveness of ZDCs,^18^^,^^19^^,^^20^ less attention has been given to understanding how full implementation and enforcement of ZDCs could potentially play out in terms of future global land use and land use change. The difficulty in predicting the potential impacts of ZDCs arises from the necessity to construct a plausible counterfactual, i.e., a scenario of what would happen in the absence of ZDCs. Such an assessment should account for global market-mediated effects that may occur in response to the implementation of ZDCs as well as for local differences in land availability, land suitability, and baseline changes in the economy. One study^7^ provided an *ex ante* assessment of the overall forest loss that may be avoided due to ZDCs in Indonesia but did not account for economy-wide effects and spillover effects in other countries. Such spillover effects have the potential to seriously undermine the effectiveness of ZDCs.^21^ A more recent study^22^ offered a comprehensive, global assessment of the amount of deforestation that may be avoided if certain economic policies are adopted by the Indonesian and Malaysian government, but they did not consider the role of ZDCs in curbing deforestation. Furthermore, both studies only assessed aggregate forest loss at the country level and did not consider spatialized deforestation outcomes within countries. As impacts depend on the spatial patterns of forest loss, more spatially explicit assessments are needed to anticipate the potential environmental benefits of the ZDCs.^23^

The objective of this paper is to make a spatial assessment of how the worldwide implementation of zero-deforestation commitments across all agricultural commodities, economic sectors, and geographies could alter the spatial configuration of oil palm and other land uses up until 2030 while accounting for expected baseline changes in the economy. The analysis will facilitate an evaluation of the extent to which fully executed ZDCs may moderate conversion of forests and other (semi) natural areas over this time period. We link the results from a computable general equilibrium model with a dynamic land use change model to project how land use may evolve under two scenarios: a business-*as*-usual (BAU) scenario assuming no implementation of ZDCs and a ZDC scenario assuming full implementation and enforcement of ZDCs across all economic sectors. The spatial distribution of oil palm and other land uses is modeled from 2014 (the most recent reference year for which global economy-wide data are available) up until 2030. The year 2030 coincides with the target year of the 2014 New York Declaration on Forests^24^; a declaration signed by more than 200 companies, governments, non-governmental organizations, and groups representing indigenous communities to end all global natural forest loss.

## Results

### Effects on oil palm area and other types of land use

**Figure 1:**
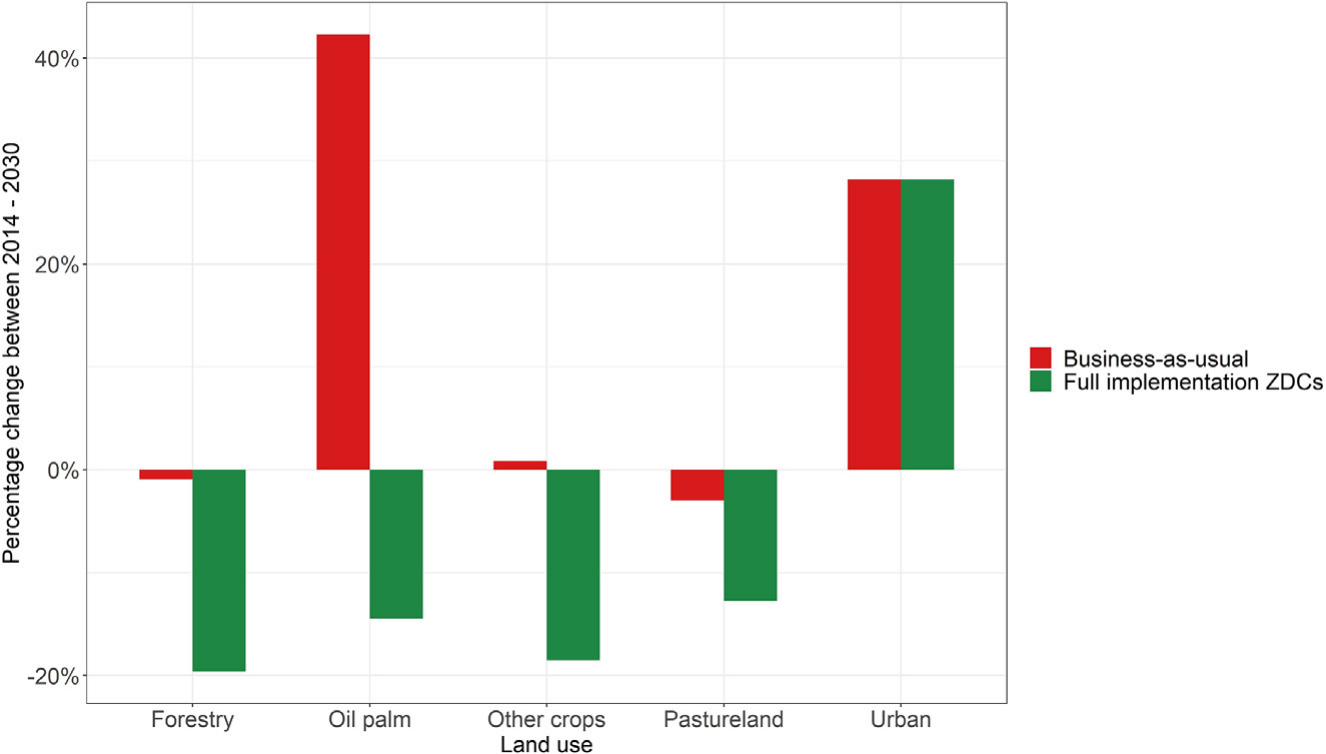
Percentage changes in land use in the oil palm-producing regions for five different types of demand relative to the area in 2014 Results are shown for two scenarios: a business-*as*-usual scenario that follows historic trends and an alternative scenario that assumes full implementation of zero-deforestation commitments (ZDCs).

**Figure 2:**
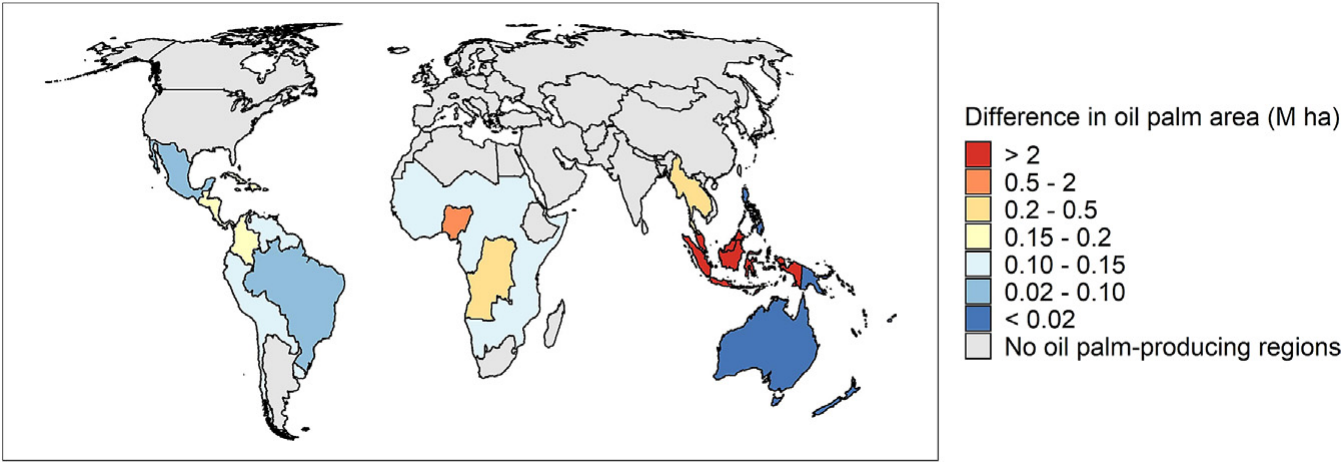
Spatial overview of the ZDCs-induced difference in oil palm area relative to the BAU scenario in 2030 In all regions, the difference represents a reduction in oil palm area.

The results of the GTAP experiments show that the global oil palm area in 2030 under the ZDC scenario is nearly 3 M ha or 14% smaller relative to the area in 2014 (see Figure 1). As the global area under oil palm is expected to expand by 42% under the BAU scenario during the same period, the worldwide implementation of ZDCs leads to a 39% smaller oil palm area relative to the BAU scenario up until 2030. The reduction in oil palm area due to ZDCs is largest in Indonesia (6.3 M ha), Malaysia (2.4 M ha), and Nigeria (0.8 M ha; see Figure 2). These results are rather sensitive to how the share of oil palm in the aggregate oil crop sector of GTAP is calculated. If it is assumed that the share of oil palm in 2030 equals the observed share within each GTAP region in 2019, the global oil palm area expands by 19% between 2014 and 2030 under the BAU scenario and reduces by 28% under the ZDC scenario over the same period.

The large contraction in oil palm area under the ZDC scenario relative to the BAU scenario is partly a result of land scarcity-induced increases in oil palm yields, which increase by 18%. Under the ZDC scenario, oil palm yields go up on average by 2.7 metric tonnes per ha or 4.6 metric tonnes per ha if regions are weighted according to their production share in 2014. Increases in oil palm yields are estimated to be highest in Malaysia, Indonesia, and Colombia (see Figure S13).

In addition to boosting yields, the ZDC-induced increase in land rental rates will partially translate into an increase in global commodity prices (see Figure S14) and hence a reduction in the demand for oil palm. Whereas oil palm production increases by 42% under the BAU scenario between 2014 and 2030, it only increases by 1% under the ZDC scenario. Thus, due to ZDCs, the global production of oil palm FFB relative to the BAU scenario is expected to decrease by 29% or 117 M metric tonnes.

The results also indicate that ZDCs will partially displace production of agricultural commodities to new areas and encourage intercrop substitutions. Production of oil crops is expected to increase by 7.4 M metric tonnes in non-oil palm-producing countries (which equates to 0.9% of the global production in 2014). It is likely that some of the production of oil palm will also be displaced to other oil palm-producing regions with more land availability. However, Figure 2 shows that none of these regions experience a net increase in oil palm due to ZDCs, which implies that any such effects are offset by the demand and yield effects. In addition to a major difference in oil palm area, total area devoted to other crops, forestry, and pastureland is also expected to decrease relative to the BAU scenario as a result of the large increases in land rents, although the percentage changes are smaller (ranging from −10% to −20%). Apart from oil palm, major reductions are expected in the area devoted to fruit and vegetables, coarse grains, and rice (see Figure S15).

These results remain largely robust if the alternative estimates of ZDC coverage are used (as described in the STAR Methods section), with the projected reduction in oil palm area relative to BAU conditions varying from 38% to 42%. Results for the other land use types are similar too, with the percentage reductions in area varying in the range of 9%–26%.

### Effects on natural areas

**Figure 3:**
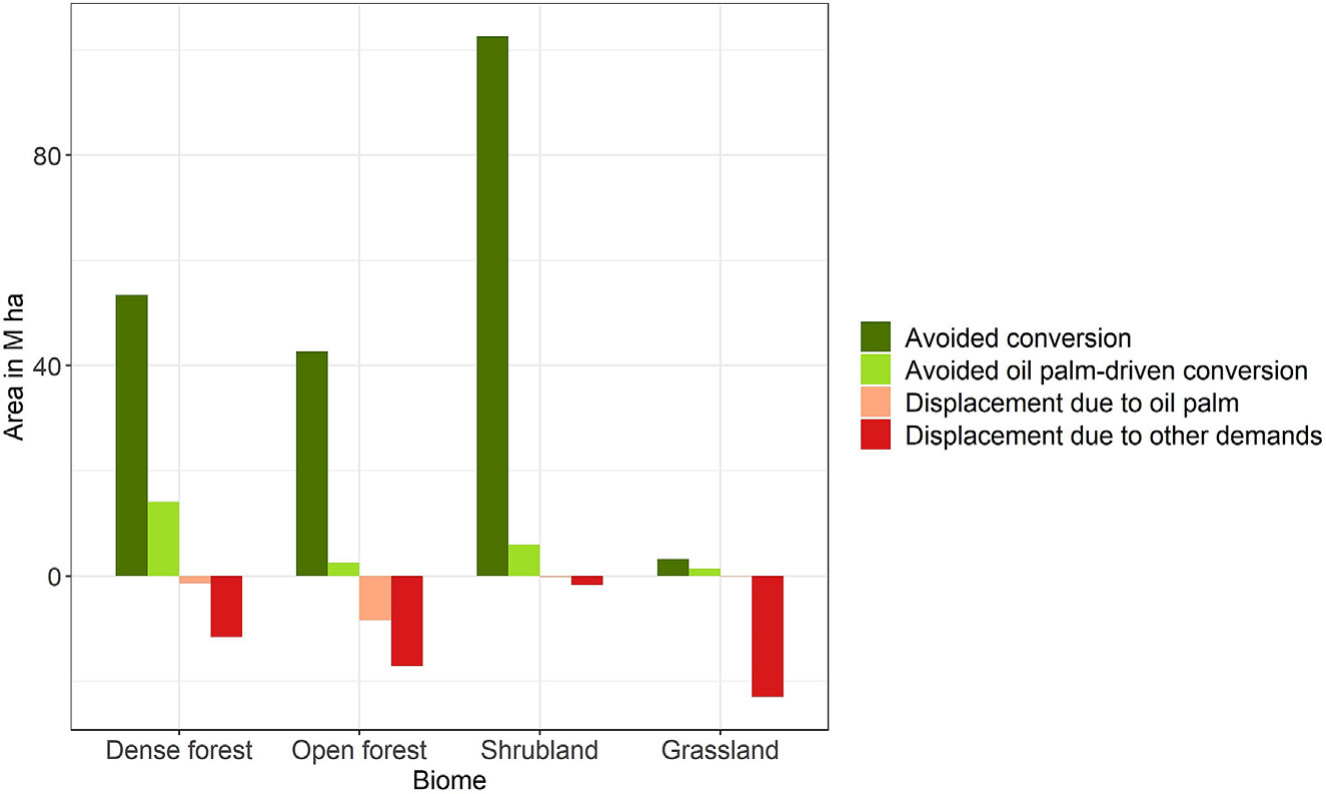
Absolute area changes as a result of ZDCs within 4 (semi-) natural biomes: dense forests, open forests, shrublands, and grasslands Green colored bars represent avoided conversions. Red colored bars represent displacement effects. Lighter colored bars indicate absolute area changes that can be directly attributed to changes in the demand for oil palm.

Due to the reduction in oil palm area and other land uses, ZDCs are estimated to avoid a lot of encroachment into (semi-) natural areas, which include dense forests, open forests, shrublands, and grasslands. Based on our downscaled land use simulations, we estimate that 53 M ha of dense forests and 43 M ha of open forests are saved from conversion, of which 26% and 6%, respectively, would otherwise have been cleared due to expanding oil palm plantations (Figure 3; note that this includes both direct and indirect oil palm-driven conversions). However, as there are large forests outside the scope of ZDCs (see Figure S11), their implementation triggers a chain of displacement effects in the simulations. Around 13 M ha of dense forests and 26 M ha of open forests are converted as a result of ZDCs, of which 11% and 33%, respectively, can be attributed to displaced oil palm plantations. Such effects are particularly large in Central Africa, Colombia, and other parts of South America. Results for shrublands are markedly different with a staggering 103 M ha of avoided conversion, of which only a small amount is displaced (2%). These effects are to a lesser extent driven by oil palm as shrublands tend to be less dominant in the main oil palm-producing regions (Figure 3). Most of the avoided conversion is in Oceania (notably Australia), where shrublands tend to be the dominant natural biome and where many of these areas are covered by ZDCs due to their high levels of above-ground biomass carbon.^25^ By comparison, only a small area of grassland is saved from conversion (3 M ha), while a much larger area is converted due to ZDCs (23 M ha, of which less than 1% can be attributed to oil palm). The reason why displacement effects for grassland are larger than for other biomes is that most grasslands fall beyond the scope of ZDCs, which is why cropland expansion tends to be redirected to grasslands. When taken in aggregate, the total extent of natural land that is saved from conversion is 217% larger than the area converted due to ZDCs. This suggests that the ZDCs are likely to deliver substantial environmental benefits within the oil palm-producing world, although there is considerable heterogeneity across regions.

## Discussion

This is the first study to provide a modeling experiment of how the worldwide implementation of zero-deforestation commitments could alter the spatial configuration of oil palm and other land uses up until 2030, assuming full adoption and enforcement across industries and regions from 2014 onward. The results suggest that under these assumptions, ZDCs are likely to bring about significant land-sparing effects. Due to increases in yields and an overall decrease in demand for oil crops, ZDCs could hypothetically induce a decrease in global oil palm area of 11 M ha or 39% relative to a BAU scenario that assumes no compliance with ZDCs and that follows historic trends. By way of comparison, FAO statistics for the period 2014–2020 report an increase of 6.8 M ha in the global extent of oil palm plantations, of which 75% occurred in the period 2014–2017. This compares with our estimate of 8.1 M ha over the period 2014–2030 under BAU conditions, which appears to be a reasonable projection in light of the flattening trend observed from 2017 onward. These results remain largely robust when using alternative estimates of the potential spatial coverage of ZDCs. A potential explanation for this is that the spatial differences between these different estimates are relatively minor after masking out areas unavailable for oil palm production, suggesting there is little additional impact when considering more ambitious forest protection scenarios. Apart from oil palm, ZDCs are also expected to induce a decrease in the total area devoted to other crops, forestry, and pastureland in the oil palm-producing world relative to the BAU scenario. Although the total area under forestry and pastureland is projected to decrease regardless of ZDCs (as would be expected given the large contraction in recent years^26^^,^^27^), both types of land use will still be major drivers of local forest loss under the BAU scenario. Overall, ZDCs are estimated to prevent the conversion of around 96 M ha of forests, of which 17% would otherwise have been converted due to expanding oil palm plantations. Notwithstanding the large land-saving effects, potential environmental benefits associated with reductions in nature loss are likely partially offset by displacement effects to natural areas that fall beyond the scope of ZDCs. Not only does this include displacement effect within oil palm-producing regions but also displacement effects to temperate regions. This suggests that temperate oil crops such as rapeseed, sunflower, and soybean are expected to partially substitute for oil palm, which is particularly likely in the case of biofuels,^28^ but also for many food applications where hydrogenation of alternative edible oils is a viable option.^29^ In addition, although yield increases help to free up crop areas for other uses, anticipated increases in the use of fertilizers, pesticides, and heavy machinery (likely needed to achieve these yield increases) could lead to larger local and global environmental impacts from existing agricultural areas.^30^ The degree to which ZDCs are to deliver environmental benefits thus partially hinges on the impact of displacement effects and the technology through which yield increases are achieved. We also find that due to land scarcity and increasing commodity prices, ZDCs are expected to depress consumption of agricultural commodities, posing potential challenges for food accessibility. What is more, imposing ZDCs on individual supply chains without considering the differential capabilities of actors to comply with them could result in the unfair market exclusion of farmers. For example, smallholder farmers may be unable to comply with deforestation-free certification schemes such as the Roundtable of Sustainable Palm Oil by virtue of the high costs involved in certification audits, lack of awareness of ZDCs, or unclear land tenure.^31^^,^^32^^,^^33^ Such concerns of distributive equity could be mitigated if ZDCs are accompanied by differentiated and locally targeted capacity-building measures. These could include financial support schemes, knowledge dissemination activities, and coordination efforts with other policy-making actors (private and public) to enhance the inclusivity of policies and to exploit synergies between ZDCs and equity. Previous research has shown that such synergies exist as poor communities stand to benefit the most from forest protection measures by virtue of the improved pollination services, forestry, and fishery yields they provide.^34^

### Limitations of the study

Our findings build on a burgeoning literature that has found that anti-deforestation policies, if implemented at large scales, could induce major environmental benefits outside targeted areas.^22^^,^^35^ However, previous studies have also found that the risk of leakage effects is much higher if adoption and implementation varies widely across space.^36^^,^^37^^,^^38^ Our results should thus be interpreted carefully as they are projected on the assumption that all industries and regions will be fully covered by ZDCs. It is likely that the anticipated land-sparing effects will be considerably diminished if large consumer markets fall beyond the scope of ZDCs, as is currently the case in China and India.^39^ Moreover, the results are rather sensitive to how the share of oil palm in the aggregate oil crop sector of GTAP is calculated, with much less oil palm expansion projected under the BAU scenario between 2014 and 2030 (19% instead of 42%) if the share of oil palm relative to other crops is assumed to remain constant rather than growing at historic rates. However, given that recent national-level statistics show a major increase in the share of oil palm over the period 2014–2019 for most oil palm-producing regions (see Figures S7A–S7C), this appears to be a less plausible assumption. Our analysis is subject to some additional uncertainties that we cannot fully address. First and foremost, it is possible that the region-specific land supply curves underpinning our analysis imply land supply elasticities that are too high for some regions. As a result, we may have overestimated the magnitude of the overall market-mediated effects of ZDCs. The land supply elasticities may be too high because data on current land prices are only available for a limited number of regions in the world.^40^ For regions with no data on land rents, it is assumed that the marginal land rents are inversely proportional to marginal yields. While this assumption may be a reasonable approximation of the land rent trajectory in many developed countries,^41^ it is less likely to hold true in developing countries with poorly functioning land markets and weakly enforced land tenure rights.^42^ A related point is that the analysis is based on a shift in the land supply asymptotes that are assumed to remain unaltered throughout the simulation period. In practice, it is more likely that such asymptotes are dynamic as they depend on annual weather fluctuations, changes in soil quality, the spatial dynamics of legally protected areas, land governance, and the degree of technological progress. A case in point is the Brazilian Cerrado, a savannah ecoregion that was largely considered unsuitable for agricultural production in the 1970s until advances in soil amendment technologies and crossbreeding transformed it into the world’s “soy basket”.^8^ It should be noted, though, that given the relatively short time period for our simulation (2014–2030), it is unlikely that such unforeseen dynamics will have a major impact on the amount of available land until 2030. A final major source of uncertainty is that our estimates of the overall impact of ZDCs are dependent on the validity of our counterfactual projections which describe what might happen in the absence of ZDCs (i.e., the BAU scenario). Although the BAU scenario largely follows the SSP2 scenario, which is considered a benchmark for baseline projections,^43^ it is likely that there will be deviations between our projections and the actual changes in oil palm area. These deviations may occur due to political economy factors that are not well predicted by a computable general equilibrium model (e.g., biofuel mandates, election cycles, and imperfect law enforcement). In addition, we may have under- or overestimated future crop yields as they are currently assumed to grow at a linear rate. Although this assumption is largely consistent with historic crop yield trajectories,^44^ future crop yields remain a key source of uncertainty as it is unknown what capital investments would be required and how this would influence the adoption rate.^45^ On a similar note, our results depend to a large extent on the factor mobility assumptions underpinning the GTAP model, especially with regard to land use type-specific conversions. While the disaggregation of each GTAP region into AEZs region in the GTAP region is an attempt to account for spatial differences in mobility of land across uses, there is still considerable heterogeneity within these units. Future research should thus explore the sensitivity of our results to alternative yield and mobility assumptions. Despite these uncertainties, our study provides a first estimate of how full implementation and enforcement of ZDCs globally may moderate the expansion of oil palm and its encroachment into forest areas. It provides a benchmark against which future estimates can be compared, for example assessments of ZDC implementation with incomplete coverage across sectors and geographies. Although it is unlikely that the coverage of ZDCs will be anywhere near 100% in the coming decade, our study provides strong quantitative evidence of the major reduction in deforestation and agricultural expansion they could deliver if they are adopted and enforced at large scale, thus resulting in potentially large environmental benefits. Given the goals of the New York Declaration on Forests and the Convention on Biological Diversity, this should motivate the international community to increase the uptake, but also the enforcement of ZDCs. Imposing mandatory due diligence rules for importers into major destination markets of deforestation-risk commodities, as currently discussed by the European Union, United Kingdom, and United States,^46^ could help accelerate progress toward making ZDCs more effective. In addition, care should be taken to engage participation of stakeholders from tropical forest countries in zero-deforestation policymaking to ensure not only the effectiveness of such policies but also the equitable conservation of tropical forests.

## STAR★Methods

### Key resources table

**Table:** 

REAGENT or RESOURCE	SOURCE	IDENTIFIER
**Deposited data**		

Land supply asymptotes for all region-specific Agro-Ecological Zones in the GTAP-AEZ database	This paper	https://www.environmentalgeography.nl/site/data-models/data/
Criteria used to identify areas unsuitable for cropland expansion	This paper	Table S1
Spatial predictor variables used in the CLUMondo simulations	This paper	Table S3
Land conversion matrix used in the CLUMondo simulations	This paper	Table S4
Conversion resistance elasticities for each land system used in the CLUMondo simulations	This paper	Table S5
List of neighborhood weight factors by land system	This paper	Table S7

**Software and algorithms**		

GTAP-AEZ	Johnson et al. (2021)^34^	https://www.gtap.agecon.purdue.edu/models/current.asp
CLUMondo	Van Asselen & Verburg (2013)^47^	https://www.environmentalgeography.nl/site/data-models/models/
R Studio 2022.02.3 + 492	RStudio: Integrated Development for R	https://posit.co/download/rstudio-desktop/

### Resource availability

#### Lead contact

Further information and requests for resources should be directed to the lead contact, Floris Leijten (florisleijten@hotmail.com).

#### Materials availability

The gridded land supply asymptotes at 1 × 1 km for all region-specific Agro-Ecological Zones in the GTAP-AEZ database under the Business-As-Usual (BAU) scenario can be downloaded from the Environmental Geography website: https://www.environmentalgeography.nl/. The gridded land supply asymptotes under the Zero-Deforestation Commitments (ZDCs) scenarios are partially based on IUCN Red List Data and therefore, cannot be redistributed without the prior written permission of IUCN: https://www.iucnredlist.org/resources/spatial-data-download.

### Method details

#### Overall approach

We took a stepwise approach to project changes in oil palm area from 2014 up to 2030, as a result of fully implemented and enforced ZDCs. We first employed a multi-commodity, multiregional comparative static computable general equilibrium model to make baseline projections of economic activity up to 2030, assuming no compliance with ZDCs. This model operates at a spatial resolution of 37 world regions, stratified by 18 global Agro-Ecological Zones (AEZs), resulting in 337 region-specific AEZs (see Figures S3 and S4), and accounts for legally protected areas using data from the World Database on Protected Areas.^48^ To account for heterogeneity in the area available for cropland expansion across the different regions, we calibrated the model with new spatially explicit estimates of region-specific supply of available land, also referred to as land supply asymptotes. We then constructed an alternative scenario that assumes full implementation of and compliance with ZDCs but otherwise equal to the BAU scenario. This scenario implies a reduction in the supply of land available for expansion equal to the areas covered by ZDCs, and hence, simulates a potential change in both the supply and demand of oil palm, and other crops, relative to the baseline scenario. The outcomes of these two scenarios were then used to model the spatial distribution of oil palm plantations within each oil palm producing region up to 2030, using a spatially explicit and dynamic land use change model, operating at a spatial resolution of 10 × 10 km. This model accounts for competition between all land uses for space and can therefore simulate displacement of land uses. Comparison of the land use configurations resulting from these two scenarios provides an indication of the potential influence of ZDCs on oil palm and other land uses up to 2030. A stylized flowchart of the methodology is shown in Figure S2.

#### Computable general equilibrium modeling

Computable general equilibrium (CGE) models are systems of mathematical equations that describe economies as a whole and the interaction among their parts.^49^ They are based on the premise of market-clearing, which occurs when the aggregate supply of goods and services equals aggregate demand. Producers are assumed to choose levels of input and output that minimize costs and consumers are assumed to maximize their utility subject to budget constraints. Policy changes can be simulated by changing variables of the model, which leads to a reallocation of labor, capital and land across sectors and geographies, until the system reaches equilibrium again. Responsiveness of producers and consumers to changes in relative prices and income is simulated by empirically calibrated elasticities of supply and demand.

In order to model the global market-mediated effects of ZDCs from 2014 up to 2030, we employed the most recent version of the Global Trade Analysis Project with differentiated Ago-Ecological Zones (GTAP-AEZ) CGE model^34^ using the most recent GTAP database.^50^ The database describes the world economy in 2014, disaggregated into 65 different sectors and 141 different regions, which we aggregated in 37 distinct regions for computational reasons (see Figure S2). Given that coverage of ZDCs across industries and regions was far from 100% in 2014, our model experiment is hypothetical as it does not inform on the actual effectiveness of ZDCs. Instead, the model experiment is designed to capture the potential effects that could be induced by ZDCs if they were fully implemented across industries and regions. To account for likely macroeconomic trends between 2014 – 2030 that are unrelated to the adoption of ZDCs, we constructed a baseline or BAU scenario for the year 2030 that is largely consistent with the second Shared Socioeconomic Pathway (SSP), a scenario where socioeconomic trends broadly follow their historical patterns.^51^^,^^52^ Following Johnson et al.,^34^ we used growth rates of real GDP, capital stock, population, unskilled and skilled labor from ECONMAP (v2.4)^53^ which are calibrated against the SSP2 scenario. Sector specific productivity growth for ruminants and non-ruminants are taken from Ludena et al. (2007).^54^ Due to lack of estimates for forest sector productivity growth, agricultural productivity growth figures are also taken from Ludena. Following Chateau et al. (2020),^55^ a 2% productivity growth gap between manufacture and service sectors is also imposed. Finally, to account for baseline growth rates in crop yields, we used data from FAOSTAT (2021) and extrapolated yields up until 2030 based on the observed linear growth rate between 1998 – 2014 (given the reference year of the GTAP database) for each agricultural commodity group within each region in the GTAP database. To avoid implausibly high yield increases, we scaled these projections using the global yield projections from the OECD-FAO Agricultural Outlook.^56^^,^^57^

A special feature of GTAP-AEZ is that, in contrast to the standard GTAP model,^58^^,^^59^ each region is further disaggregated into spatially heterogeneous land endowments or AEZs. These AEZs are determined based on climatic zones (tropical, temporal and boreal) and the length of the growing period (6 x 60-day intervals, see Lee et al., 2005^60^), resulting in 18 different AEZs that may intersect multiple countries (see Figure S4). Within each region-specific AEZ (337 in total), the supply of available land is modeled using an empirically calibrated asymptotic curve specifying the relationship between land supply and the real land rental rate.^40^^,^^41^ The land supply curves are predicated on the assumptions that expansion occurs within the most productive areas that are still available and that the land rental rate monotonically increases whenever the supply of available land decreases (see Figure S6). The maximum area available for expansion within each region-specific AEZ is constrained by a prespecified land supply asymptote. As land supply is critical in our scenarios, spatial differences in land availability need to be accounted for in the most accurate way. Therefore, we updated GTAP-AEZ by including new estimates of the land supply asymptotes for each region-specific AEZ, thereby better accounting for the constraints to land availability defined by earlier studies.^61^^,^^62^

A challenge for our model experiment is that the most recent GTAP database aggregates all oil crops (including oil palm) into one sector. Hence, to distinguish between oil palm and other oil crops in our analysis of GTAP outcomes, we used data from FAOSTAT (2021) on the area and production volumes of oil palm and other oil crops over the period 1998–2019 and made region-specific projections with respect to the oil palm share up until 2030. We considered 4 alternative approaches to project the share of oil palm and used the period 2015–2019 to evaluate the accuracy of each approach within each region. Accuracy was measured through the Root Mean Square Error (RMSE) of the predictions. For each region, we identified the approach with the lowest RMSE during the period 2015–2019 and used it to make projections up until 2030, provided that the RMSE in the period 1998–2014 was not unreasonably high (i.e., more than 2 times the size of the approach with the lowest RMSE during the period 1998–2014). The 4 different approaches were based on linear and quadratic extrapolations of the observed share over the period 1998–2014, constant value extrapolations using the observed share in 2014, and constant value extrapolations using the observed average share in the period 2010–2014 (see Figures S7A–S7C for an overview of the different approaches by region). To explore the sensitivity of our results to the choice of attribution method, we present alternative results using the observed share of oil palm in 2019 as a parameter to attribute changes in the oil crop sector to oil palm.

#### Land supply asymptotes

To estimate the extent of area that is potentially available for cropland expansion within each region-specific Agro-Ecological Zone (AEZ), we took three steps (see Figure S8 for a flowchart of the methodology). We first identified areas where cropland could possibly expand given certain biophysical, socio-economic, and institutional constraints through a residual approach. This means, we computed the total area within each region-specific AEZ after excluding areas already under cultivation, areas biophysically unsuitable for cropland cultivation, legally protected areas, rough terrains, and urban areas (see Table S1). To harmonize the different input data, all data were resampled to a 1 km^2^ grid.

We used data from the European Space Agency Climate Change Initiative^63^; for the year 2014 to identify existing cropland areas and urban areas. Areas classified as “Cropland, rainfed”, “Cropland, irrigated or post-flooding” and “Urban areas” were assumed to be unavailable for expansion. The first two of these three classes do not cover all cropland areas as some cropland areas in the ESA-CCI database are subsumed under two mosaic classes (“Mosaic cropland (>50%) / natural vegetation (<50%)” and “Mosaic natural vegetation (>50%) / cropland (<50%)”). Following Liu et al. (2018),^64^ we therefore assumed a cropland fraction of 58% and 38% for these two mosaic classes, respectively. To identify areas biophysically unsuitable for overall cropland expansion, we used data from Zabel et al. (2014).^65^ These data incorporate suitability estimates for the 16 most important food and energy crops and account for the potential impact of climate change under SRES A1B conditions. Under this scenario, economies will rapidly grow, population growth will be small and there will be a rapid introduction of new and more efficient technologies. Areas classified as unsuitable were assumed to be unavailable for expansion. Furthermore, we excluded areas that are legally protected.^66^ Although it has been recognized that encroachment may still occur within these areas,^67^ it is unlikely that these areas represent future hotspots of agricultural expansion.^68^ Finally, we excluded rough terrains (here defined as areas with steep slopes) as these areas are less likely to be developed.^69^ The extent to which rough terrains can be cultivated depends, however, on the degree of agricultural mechanization,^70^ which varies across space. Therefore, in an attempt to control for the spatially varying levels of agricultural mechanization, we took a region-specific approach to identify areas that are too steep to convert into cropland. Within each of the 37 regions in the GTAP database, we applied a slope threshold, based on the top 5% slope values within existing cropland areas. Slope data were sourced from Lloyd (2016).^71^ As this resulted in unreasonably pessimistic slope thresholds in areas that are dominated by flat terrains, we imposed a minimum threshold of 10 degrees. To account for tiny landscape features that inhibit cropland expansion but cannot be detected at a 1 km^2^ resolution (e.g., roads, rocky outcrops, water infrastructure, hedgerows, buildings etc.) we multiplied the extent of the remaining area of each region-specific AEZ by 0.85. This parameter is based on Verburg et al. (2009),^72^ who estimated that across different agriculturally dominated landscapes about 15% of the area is not used for crop cultivation. This approach for calculating available land may over- or underestimate the extent of the available area that can be harvested as it does not account for multiple cropping and fallow systems.^73^^,^^74^ Therefore, for each AEZ, we further multiplied the extent of the total area available for expansion by the estimated region-specific multiple cropping intensity in 2014. This approach assumes that the multiple-cropping intensity within each region-specific AEZ remains constant between 2014 – 2030. To reduce the influence of outliers (areas with either extremely high or low cropping intensities, see Figure S9), we constrained the cropping intensities to lie in the range 0.5–2.0. Formally, this means we computed the area available for cropland expansion within each region-specific AEZ as follows:

(Equation 1) $$Available\;area_{i}=\left\{ \begin{array}{c} \left(TA_{i}-UA_{i}\right)*0.5\\ \left(TA_{i}-UA_{i}\right)*CI_{j}\\ \left(TA_{i}-UA_{i}\right)*2 \end{array}\right.\begin{array}{c} if\;CI_{j}<0.5\\ if\;0.5\leq CI_{j}\leq 2\text{,}\\ ifCI_{j}>2 \end{array}$$

where $Available\;area_{i}$ denotes the available area within region-specific AEZ *i* in hectares; $TA_{i}$ denotes the total terrestrial area in hectares; $UA_{i}$ denotes the total area unsuitable for cropland expansion in hectares; and $CI_{i}$ denotes the cropping intensity. Cropping intensities were estimated by computing for each region-specific AEZ the ratio of the harvested area of all crops in the GTAP database^50^ to the total extent of cultivated area, based on the 10 arc-seconds resolution European Space Agency - Climate Change Initiative (ESA-CCI) satellite-based land cover map for the year 2014 (see Table S1 of for further information). Finally, we summed the total harvestable area available for expansion and the total area harvested in 2014 to arrive at an estimate of the land supply asymptote for each region-specific AEZ (see Figure S10A).

#### Implementation of zero-deforestation commitments

Implementing ZDCs involves reducing the (forest) area available for cropland expansion and hence, shifting the land supply asymptote to the left (see Figure S6). There are large uncertainties regarding the spatial coverage of ZDCs as the uptake and specificity of ZDCs varies across individual firms, commodities, and regions.^75^ Nevertheless, common criteria outlined in many ZDCs are the protection of High Conservation Value Forests (HCVF) and High Carbon Stock Forests (HCSF). At least 78% of the palm oil refining capacity in Indonesia and Malaysia is covered by commitments which include these criteria^17^ and the uptake of similar commitments in other industries and regions is on the rise.^76^^,^^77^ We used data from Leijten et al. (2020)^25^ on the likely spatial distribution of HCVFs and HCSFs to delineate areas covered by ZDCs (see Figure S11). We focused on a middle-of-the-road estimate, which includes all tropical peatland forests and all forests with at least two overlapping HCVF categories or with at least 75 tonnes of carbon per hectare (t C ha^−1^) if located in the tropics (see Leijten et al. for further details). Updated estimates of the land supply asymptotes that account for the coverage of ZDCs are presented in Figure S10B. To explore the robustness of our results, we present alternative results using the top and bottom range estimates of the likely spatial extent of HCVFs and HCSFs. The low estimate (i.e., least protective) is based on three overlapping HCVF categories with a 75 t C ha^−1^ threshold while the upper estimate only requires one HCVF category with a 35 t C ha^−1^ threshold.

#### Spatial land use modeling

We used the GTAP-AEZ output as input for a dynamic land use model to simulate the spatial dynamics of oil palm and other land systems within all oil palm producing regions.

##### CLUMondo

CLUMondo is a spatially explicit and recursive dynamic land change model that can be used to simulate the spatial dynamics of land systems.^47^^,^^78^ It is an open-source model written in C++ that can be downloaded from: https://www.environmentalgeography.nl/site/data-models/models/. One of the unique features of the model is that it incorporates multifunctional land systems, i.e., land systems that provide multiple types of goods and services. To simulate future land use configurations, the model uses an iterative procedure where grid cells are allocated to the land system with the highest transition potential constrained by the overall regional production levels as determined by CGE models. Transition potentials represent the potential of a grid cell to be converted into a certain land system and are calculated for each individual land system though the following formula:

(Equation 2) $$Ptrans_{i,t,LS}=Ploc_{i,t,LS}+Pres_{LS}+Pcomp_{t,LS}+Pneigh_{i,t,LS}$$

where $Ptrans_{t,i,LS}$ represents the overall transition potential to land system *LS* in grid cell *i* at time *t*, $Ploc_{i,t,LS}$ represents the local suitability of land system *LS*, $Pres_{LS}$ represents the conversion resistance of land system *LS*, proxying conversion and investment costs as well as cultural attachments to the current land use, $Pcomp_{t,LS}$ represents the relative competitive advantage of land system *LS*, determined by the demand for the different products/services delivered by the land system and $Pneigh_{i,t,LS}$ denotes the neighborhood influence, representing agglomeration processes, for example due to economies of scale (2.5.5). By iteratively updating $Pcomp_{t,LS}$, the model constructs a spatial configuration of land use such that meets the aggregate demand of goods and services within each region, here represented by the demand projections of the GTAP-AEZ model. Further details on the allocation procedure can be found in Van Asselen and Verburg (2013).^47^

To simulate the likely future spatial distribution of oil palm (given the GTAP-AEZ projections), we first created a 10 × 10 km land use map for the year 2014, the reference year of the GTAP-AEZ database. The land use map was primarily based on the 2014 ESA-CCI land cover map,^63^ which we reclassified into 8 distinct land cover classes (see Table S2). To distinguish oil palm producing systems from other systems, we overlaid the resulting land cover map with a remotely sensed oil palm map from Descals et al. (2021),^79^ aggregated to a 10 × 10 km resolution. We classified all grid cells with at least 50% oil palm cover as oil palm areas. Grid cells with 10–50% oil palm cover were classified as oil palm mosaics, which in addition to oil palm, produce a variety of other agricultural commodities (see below). GTAP regions with no oil palm areas or oil palm mosaic (according to the Descals map) were excluded from the analysis.

Demand for all other agricultural commodities is supplied by either cropland areas or cropland mosaics (which, like oil palm mosaics, represent a mixture of different land uses). As these two cropland categories vary in their agricultural intensity across space, we distinguished between high yielding systems, medium yielding systems, and low yielding (or extensive) systems. To classify cropland areas and cropland mosaics into these three different yielding systems, we overlaid the land use map with spatially explicit data (10 × 10 km) on the yields of all agricultural crops from the International Food Policy Research Institute (2019) and divided the data into quantiles. More specifically, we computed the sum of all yields within each pixel and selected the three highest quintiles (i.e., the three highest cut-off values that divide the distribution into five equal parts) to differentiate between high yielding, medium yielding and low yielding systems, respectively.

Finally, to distinguish grasslands that are intensively grazed from other grasslands, we used spatially explicit data (10 × 10 km) on the distribution of cattle in 2010 from Gilbert et al. (2018).^80^ Grasslands within the top octile (12.5%) of the cattle density distribution were assumed to be intensively grazed. Figure S12 shows the resulting land use map. GTAP-regions with no or barely any oil palm area were excluded from the CLUMondo simulations. This includes China and Madagascar, which both accounted for less than 0.23% of global oil palm area in 2014.^1^

##### Local suitability

Local suitability ($Ploc_{i,t,LS}$; see Equation 2) of the different land systems was assessed based on empirically quantified relations between land use patterns and several explanatory variables, using a logistic regression analysis for each land system within each oil palm producing region. Table S3 lists all 44 predictor variables used in the regression analysis. Model selection was done through a backward model selection procedure based on Akaike’s Information Criterion. To avoid multicollinearity, predictor variables with intercorrelations exceeding 0.8 were removed from the analysis. To allow for dynamic changes in local suitability, we used spatiotemporal predictions of total and rural population density up to 2030 from CIESIN (2018).^81^ The data were linearly interpolated for years for which no predictions were available.

##### Conversion resistance

For each land system in each region, a parameter was specified that captures the degree to which it is resistant to any type of land system conversion ($Pres_{LS}$, in Equation 2. Land systems that involve large capital investments are typically more resistant to change.^82^ Oil palm plantations typically require a large amount of capital investment, especially since they only start bearing fruit in the third or fourth year after establishment.^83^ For that reason, we assumed that oil palm plantations tend to be relatively resistant to change, whereas land systems requiring little capital investment (e.g., extensive croplands) were assumed to be more reversible (less resistant to change once established at a location) and thus assigned a lower conversion resistance factor (see Table S5). In addition to conversion resistance, the spatiotemporal dynamics of the simulation are also influenced by a prespecified land conversion matrix that stipulates the type of land system conversions that are allowed and how long it takes before such conversions may take place. Given the time lag between the establishment of an oil palm plantation and the yielding period, the land conversion matrix was specified such that oil palm plantations can only be established after three years with stable land use. Finally, we also assumed, based on the estimates of Liebsch et al. (2008),^84^ that after a period of 30 years, open forest may mature into dense forest. In the absence of detailed information on the history of each land system, we assigned a random number to all pixels indicating the number of years that the land system is already in place. All other land conversion rules developed for this analysis can be found in Tables S4 and S5.

##### Relative competitive advantage

The relative competitive advantage ($Pcomp_{t,LS}$ in Equation 2) of a land system in a certain region is determined through an iterative procedure in which the provided goods and services are compared to the total demand for those goods and services in the same region. Drawing on the GTAP-AEZ projections, we distinguished between five different types of demand: forestry, oil palm, other agricultural commodities, pastureland and urban areas; each of which is supplied by at least one land system (see Table S6). As our GTAP-AEZ results only provide predictions for the year 2030, we interpolated the annual changes in demand between 2014 – 2030 based on the compounded growth rate of each type of demand. To ensure consistency with the GTAP projections, both in terms of area allocated to crops as well as to production volumes, the allocation was constrained by both the hectarage and the production volume. Given these constraints, the land allocation procedure determines the area and distribution of production in terms of low, medium and high intensity land systems. Since GTAP-AEZ does not solve for the amount of urban land in a region, we used global projections of future urban land expansion for the years 2020 and 2030 that are consistent with the SSP2 scenario from Chen et al. (2020).^85^ Future expansion patterns of urban land were assumed to be independent of the implementation of ZDCs. To determine the quantities of the different goods and services that each land system provides per unit area, we took two steps. First, we used the aggregate demand values for the year 2014 to compute the average yield per grid cell across all the yielding land systems. Second, to calibrate the relative yields of the different land systems, we used several spatially explicit datasets on yields or production area ratios from the International Food Policy Research Institute (2019)^86^ and computed the relative yields of the different systems. Spatially explicit data on production forests and grazing grasslands were sourced from Schulze et al. (2019)^87^ and (Gilbert et al. (2018).^80^ Finally, to attribute changes in land use to changes in oil palm demand, we compared the outcomes of our BAU and ZDC CLUMondo runs with two counterfactual scenarios (one for each scenario) that keep demand for oil palm constant until 2030 but are otherwise equal to the BAU scenario. This approach ensures that the oil palm-driven changes in land use can be distinguished from the other non-oil-palm driven changes in land use.

##### Neighborhood influence

Land use changes are often influenced by the spatial configuration of land use in neighboring areas. For example, it has been found that the strongest determinant of oil palm expansion in Malaysia is proximity to previously existing plantations.^88^ This is especially important for oil palm as several plantations often deliver to one single mill, which has to be in close proximity. To account for such neighborhood influences ($Pneigh_{i,t,LS}\text{,}$ in Equation 2), we used a 3 x 3 kernel function to adjust the transition potential in each grid cell for each land system, depending on the land system configuration in neighboring grid cells. The magnitude of the neighborhood effect was determined by a set of weight factors (see Table S7) and the fraction of the neighborhood that is occupied by each land system. Given that some land systems produce multiple goods (e.g., oil palm mosaics produce both oil palm and other crops), we scaled the weight factors depending on the average area composition of each land system. Finally, in light of the empirically supported theory that intensification is more likely to occur if land availability is scarce,^89^^,^^90^^,^^91^ we followed Van Asselen and Verburg (2013)^47^ by implementing a function that promotes cropland intensification under limited land availability and extensification under high land availability. Land availability within each 3 x 3 kernel was measured based on the land supply asymptotes described above.

## Data Availability

All the source data used for this study are publicly available (see Tables S1 and S3). All code used to analyze the data in this paper is available from the lead contact upon reasonable request.

## References

[1] FAOSTAT (2021). http://www.fao.org/faostat/en/#data/QC.

[2] Corley R.H.V., Tinker P.B. (2015).

[3] Woittiez L.S., van Wijk M.T., Slingerland M., van Noordwijk M., Giller K.E. (2017). Yield gaps in oil palm: a quantitative review of contributing factors. Eur. J. Agron..

[4] Carrasco L.R., Larrosa C., Milner-Gulland E.J., Edwards D.P. (2014). A double-edged sword for tropical forests: contrary to expectation, high-yield tropical crops may cause forest loss in the tropics. Science.

[5] Bentivoglio D., Finco A., Bucci G., Zolin M.B. (2018). Asian palm oil production and European vegetable oil market: what can we learn in terms of sustainability?. Asian Nations and Multinationals.

[6] OECD, FAO (2020). OECD-FAO AGRICULTURAL OUTLOOK 2020-2029.

[7] Mosnier A., Boere E., Reumann A., Yowargana P., Pirker J., Havlík P. (2017). Palm Oil and Likely Futures Plantations in Indonesia.

[8] Byerlee D., Falcon W.P., Naylor R.L. (2016).

[9] Qaim M., Sibhatu K.T., Siregar H., Grass I. (2020). Environmental, economic, and social consequences of the oil palm boom. Annu. Rev. Resour. Economics.

[10] Vijay V., Pimm S.L., Jenkins C.N., Smith S.J. (2016). The impacts of oil palm on recent deforestation and biodiversity loss. PLoS One.

[11] Harmen Smit H., Meijaard E., van der Laan C., Mantel S., Budiman A., Verweij P. (2013). Breaking the link between environmental degradation and oil palm expansion: a method for enabling sustainable oil palm expansion. PLoS One.

[12] Pirker J., Mosnier A., Kraxner F., Havlík P., Obersteiner M. (2016). What are the limits to oil palm expansion?. Global Environ. Change.

[13] Furumo P.R., Aide T.M. (2017). Characterizing commercial oil palm expansion in Latin America: land use change and trade. Environ. Res. Lett..

[14] Brandão F., Schoneveld G., Pacheco P., Vieira I., Piraux M., Mota D. (2021). The challenge of reconciling conservation and development in the tropics: lessons from Brazil’s oil palm governance model. World Dev..

[15] Lambin E.F., Gibbs H.K., Heilmayr R., Carlson K.M., Fleck L.C., Garrett R.D., le Polain De Waroux Y., McDermott C.L., McLaughlin D., Newton P. (2018). The role of supply-chain initiatives in reducing deforestation. Nat. Clim. Change.

[16] Trase (2020).

[17] ten Kate A., Kuepper B., Piotrowski M. (2020). NDPE Policies Cover 83% of Palm Oil Refineries; Implementation at 78%.

[18] Leijten F., dos Reis T.N., Sim S., Verburg P.H., Meyfroidt P. (2022). The influence of company sourcing patterns on the adoption and effectiveness of zero-deforestation commitments in Brazil’s soy supply chain. Environ. Sci. Pol..

[19] Heilmayr R., Rausch L.L., Munger J., Gibbs H.K. (2020). Brazil’s Amazon Soy Moratorium reduced deforestation. Nat. Food.

[20] zu Ermgassen E., Ayre B., Godar J., Bastos Lima M.G., Bauch S., Garrett R., Green J., Lathuillière M.J., Löfgren P., MacFarquhar C. (2020). Using supply chain data to monitor zero deforestation commitments: an assessment of progress in the Brazilian soy sector. Environ. Res. Lett..

[21] Meyfroidt P., Lambin E.F. (2009). Forest transition in Vietnam and displacement of deforestation abroad. Proc. Natl. Acad. Sci. USA.

[22] Taheripour F., Hertel T.W., Ramankutty N. (2019). Market-mediated responses confound policies to limit deforestation from oil palm expansion in Malaysia and Indonesia. Proc. Natl. Acad. Sci. USA.

[23] Lam W.Y., Kulak M., Sim S., King H., Huijbregts M.A.J., Chaplin-Kramer R. (2019). Greenhouse gas footprints of palm oil production in Indonesia over space and time. Sci. Total Environ..

[24] Schulte I., Streck C., Roe S. (2019).

[25] Leijten F., Sim S., King H., Verburg P. (2020).

[26] FAO (2016).

[27] FAOSTAT (2021).

[28] Santeramo F.G., di Gioia L., Lamonaca E. (2021). Price responsiveness of supply and acreage in the EU vegetable oil markets: policy implications. Land Use Pol..

[29] Parsons S., Raikova S., Chuck C.J. (2020). The viability and desirability of replacing palm oil. Nat. Sustain..

[30] Pellegrini P., Fernández R.J. (2018). Crop intensification, land use, and on-farm energy-use efficiency during the worldwide spread of the green revolution. Proc. Natl. Acad. Sci. USA.

[31] Grabs J., Cammelli F., Levy S.A., Garrett R.D. (2021). Designing effective and equitable zero-deforestation supply chain policies. Global Environ. Change.

[32] Lyons-White J., Pollard E.H., Catalano A.S., Knight A.T. (2020). Rethinking zero deforestation beyond 2020 to more equitably and effectively conserve tropical forests. One Earth.

[33] McDermott C.L., Montana J., Bennett A., Gueiros C., Hamilton R., Hirons M., Maguire-Rajpaul V.A., Parry E., Picot L. (2022). Transforming land use governance: global targets without equity miss the mark. Env. Pol. Gov..

[34] Johnson J.A., Ruta G., Baldos U., Cervigni R., Chonabayashi S., Corong E., Gavryliuk O., Gerber J., Hertel T., Nootenboom C. (2021).

[35] Overmars K.P., Stehfest E., Tabeau A., van Meijl H., Beltrán A.M., Kram T. (2014). Estimating the opportunity costs of reducing carbon dioxide emissions via avoided deforestation, using integrated assessment modelling. Land Use Pol..

[36] Busch J., Amarjargal O., Taheripour F., Austin K.G., Siregar R.N., Koenig K., Hertel T.W. (2022). Effects of demand-side restrictions on high-deforestation palm oil in Europe on deforestation and emissions in Indonesia. Environ. Res. Lett..

[37] Haddad S., Britz W., Börner J. (2019). Economic impacts and land use change from increasing demand for forest products in the European bioeconomy: a general equilibrium based sensitivity analysis. Forests.

[38] Ingalls M.L., Meyfroidt P., To P.X., Kenney-Lazar M., Epprecht M. (2018). The transboundary displacement of deforestation under REDD+: problematic intersections between the trade of forest-risk commodities and land grabbing in the Mekong region. Global Environ. Change.

[39] Ingram V., Behagel J., Mammadova A., Verschuur X. (2020).

[40] Woltjer G.B., Kuiper M.H. (2014).

[41] Eickhout B., van Meijl H., Tabeau A., Stehfest E. (2009). Economic Analysis of Land Use in Global Climate Change Policy.

[42] Bah E.M., Faye I., Geh Z.F. (2018). Housing Market Dynamics in Africa.

[43] Crespo Cuaresma J., Fengler W., Kharas H., Bekhtiar K., Brottrager M., Hofer M. (2018). Will the Sustainable Development Goals be fulfilled? Assessing present and future global poverty. Palgrave Commun..

[44] Grassini P., Eskridge K.M., Cassman K.G. (2013). Distinguishing between yield advances and yield plateaus in historical crop production trends. Nat. Commun..

[45] Plevin R.J., Beckman J., Golub A.A., Witcover J., O’Hare M. (2015). Carbon accounting and economic model uncertainty of emissions from biofuels-induced land use change. Environ. Sci. Technol..

[46] Mongabay (2022).

[47] Van Asselen S., Verburg P.H. (2013). Land cover change or land-use intensification: simulating land system change with a global-scale land change model. Global Change Biol..

[48] UNEP-WCMC, IUCN (2022). Protected planet: the world database on protected areas (WDPA) and world database on other effective area-based conservation measures (WD-OECM) [Online]. http://www.protectedplanet.net.

[49] Burfisher M.E. (2011).

[50] Aguiar A., Chepeliev M., Corong E.L., McDougall R., van der Mensbrugghe D. (2019). The GTAP data base: version 10. J. Glob. Econ. Anal..

[51] van Vuuren D.P., Stehfest E., Gernaat D.E., Doelman J.C., van den Berg M., Harmsen M., de Boer H.S., Bouwman L.F., Daioglou V., Edelenbosch O.Y. (2017). Energy, land-use and greenhouse gas emissions trajectories under a green growth paradigm. Global Environ. Change.

[52] O’Neill B.C., Kriegler E., Riahi K., Ebi K.L., Hallegatte S., Carter T.R., Mathur R., van Vuuren D.P., Kriegler E., Riahi K. (2014). A new scenario framework for climate change research: the concept of shared socioeconomic pathways. Clim. Change.

[53] Fouré J., Bénassy-Quéré A., Fontagné L. (2013). Modelling the world economy at the 2050 horizon. Econ. Transit..

[54] Ludena C.E., Hertel T.W., Preckel P.v., Foster K., Nin A. (2007). Productivity growth and convergence in crop, ruminant, and nonruminant production: measurement and forecasts. Agric. Econ..

[55] Chateau J., Corong E., Lanzi E., Carrico C., Fouré J., Laborde D. (2020). Characterizing supply-side drivers of structural change in the construction of economic baseline projections. J. Glob. Econ. Anal..

[56] OECD-FAO (2018).

[57] OECD/FAO (2021).

[58] Hertel T.W. (1997).

[59] Corong E., Thomas H., Robert M., Tsigas M., van der Mensbrugghe D. (2017). The standard GTAP model, version 7. J. Glob. Econ. Anal..

[60] Lee H.L., Hertel T.W., Sohngen B., Ramankutty N. (2005).

[61] Eitelberg D.A., van Vliet J., Verburg P.H. (2015). A review of global potentially available cropland estimates and their consequences for model-based assessments. Global Change Biol..

[62] Lambin E.F., Gibbs H.K., Ferreira L., Grau R., Mayaux P., Meyfroidt P., Morton D.C., Rudel T.K., Gasparri I., Munger J. (2013). Estimating the world’s potentially available cropland using a bottom-up approach. Global Environ. Change.

[63] Defourny P., Bontemps S., Lamarche C., Brockmann C., Boettcher M., Wevers J., Kirches G., Santoro M., ESA (2017).

[64] Liu X., Yu L., Li W., Peng D., Zhong L., Li L., Xin Q., Lu H., Yu C., Gong P. (2018). Comparison of country-level cropland areas between ESA-CCI land cover maps and FAOSTAT data. Int. J. Rem. Sens..

[65] Zabel F., Putzenlechner B., Mauser W. (2014). Global agricultural land resources - a high resolution suitability evaluation and its perspectives until 2100 under climate change conditions. PLoS One.

[66] UNEP-WCMC, IUCN (2018). Protected planet: the world database on protected areas (WDPA)/the global database on protected areas management effectiveness (GD-PAME)] [On-line]. https://www.protectedplanet.net/.

[67] Wolf C., Levi T., Ripple W.J., Zárrate-Charry D.A., Betts M.G. (2021). A forest loss report card for the world’s protected areas. Nat. Ecol. Evol..

[68] Molotoks A., Stehfest E., Doelman J., Albanito F., Fitton N., Dawson T.P., Smith P. (2018). Global projections of future cropland expansion to 2050 and direct impacts on biodiversity and carbon storage. Global Change Biol..

[69] Busch J., Ferretti-Gallon K. (2017). What drives deforestation and what stops it? A meta-analysis. Rev. Environ. Econ. Pol..

[70] Jasinski E., Morton D., DeFries R., Shimabukuro Y., Anderson L., Hansen M. (2005). Physical landscape correlates of the expansion of mechanized agriculture in Mato Grosso, Brazil. Earth Interact..

[71] Lloyd C.T. (2016). WorldPop Archive global gridded spatial datasets. Version Alpha 0.9. 100m base topography (tiled). Harvard Dataverse.

[72] Verburg P.H., van de Steeg J., Veldkamp A., Willemen L. (2009). From land cover change to land function dynamics: a major challenge to improve land characterization. J. Environ. Manag..

[73] Waha K., Dietrich J.P., Portmann F.T., Siebert S., Thornton P.K., Bondeau A., Herrero M. (2020). Multiple cropping systems of the world and the potential for increasing cropping intensity. Global Environ. Change.

[74] Siebert S., Portmann F.T., Döll P. (2010). Global patterns of cropland use intensity. Rem. Sens..

[75] Jopke P., Schoneveld G.C. (2018).

[76] Cheyns E., Silva-Castañeda L., Aubert P.-M. (2019). Missing the forest for the data? Conflicting valuations of the forest and cultivable lands. Land use policy In press.

[77] Areendran G., Sahana M., Raj K., Kumar R., Sivadas A., Kumar A., Deb S., Gupta V.D. (2020). A systematic review on high conservation value assessment (HCVs): challenges and framework for future research on conservation strategy. Sci. Total Environ..

[78] Schulze K., Malek Ž., Verburg P.H. (2021). How will land degradation neutrality change future land system patterns? A scenario simulation study. Environ. Sci. Pol..

[79] Descals A., Wich S., Meijaard E., Gaveau D.L.A., Peedell S., Szantoi Z. (2021). High-resolution global map of smallholder and industrial closed-canopy oil palm plantations. Earth Syst. Sci. Data Discuss..

[80] Gilbert M., Nicolas G., Cinardi G., Van Boeckel T.P., Vanwambeke S.O., Wint G.R.W., Robinson T.P. (2018). Global distribution data for cattle, buffaloes, horses, sheep, goats, pigs, chickens and ducks in 2010. Sci. Data.

[81] CIESIN (2018).

[82] Mellino S., Buonocore E., Ulgiati S. (2015). The worth of land use: a GIS-emergy evaluation of natural and human-made capital. Sci. Total Environ..

[83] Rist L., Feintrenie L., Levang P. (2010). The livelihood impacts of oil palm: smallholders in Indonesia. Biodivers. Conserv..

[84] Liebsch D., Marques M.C., Goldenberg R. (2008). How long does the Atlantic Rain Forest take to recover after a disturbance? Changes in species composition and ecological features during secondary succession. Biol. Conserv..

[85] Chen G., Li X., Liu X., Chen Y., Liang X., Leng J., Xu X., Liao W., Qiu Y., Wu Q., Huang K. (2020). Global projections of future urban land expansion under shared socioeconomic pathways. Nat. Commun..

[86] International Food Policy Research Institute (2019).

[87] Schulze K., Malek Ž., Verburg P.H. (2019). Towards better mapping of forest management patterns: a global allocation approach. For. Ecol. Manage..

[88] Shevade V.S., Loboda T.v. (2019). Oil palm plantations in Peninsular Malaysia: determinants and constraints on expansion. PLoS One.

[89] Kyalo Willy D., Muyanga M., Jayne T. (2019). Can economic and environmental benefits associated with agricultural intensification be sustained at high population densities? A farm level empirical analysis. Land Use Pol..

[90] Hadush M., Holden S.T., Tilahun M. (2019). Does population pressure induce farm intensification? Empirical evidence from Tigrai Region, Ethiopia. Agric. Econ..

[91] Boserup E. (1965).

