# Supplementary material for: Projecting global oil palm expansion under zero-deforestation commitments: Direct and indirect land use change impacts
Source: iScience. 2023 May 26;26(6):106971. doi: 10.1016/j.isci.2023.106971 (PMC10275959; doi:10.1016/j.isci.2023.106971)
Supplement: Document S1. Figures S1–S15 and Tables S1–S7 [file mmc1.pdf]

iScience, Volume *26*

Supplemental information

Projecting global oil palm expansion under zero-deforestation commitments: Direct and indirect land use change impacts

Floris Leijten, Uris Lantz C Baldos, Justin A. Johnson, Sarah Sim, and Peter H. Verburg

Supplemental information

Projecting global oil palm expansion under zero-deforestation commitments: direct and indirect land use change impacts

Authors and affiliations:

Floris Leijten[1,6], Uris Lantz C Baldos[2], Justin A. Johnson[3], Sarah Sim[4], Peter H Verburg[1,5]

1 - Environmental Geography Group, Institute for Environmental Studies (IVM), Vrije Universiteit Amsterdam, Amsterdam 1081HV, The Netherlands

2 - Department of Agricultural Economics, Purdue University, 403 West State Street, West Lafayette, IN 47907, United States of America

3 - Institute on the Environment, University of Minnesota, Saint Paul, MN 55108, United States of America

4 - Safety and Environmental Assurance Centre, Unilever R&D, Colworth Science Park, Sharnbrook, Bedfordshire, United Kingdom.

5 - Swiss Federal Institute for Forest, Snow and Landscape Research, Birmensdorf, Switzerland.

6 – Lead contact

Corresponding author: Floris Leijten· florisleijten@hotmail.com

---

Appendix A – Figures

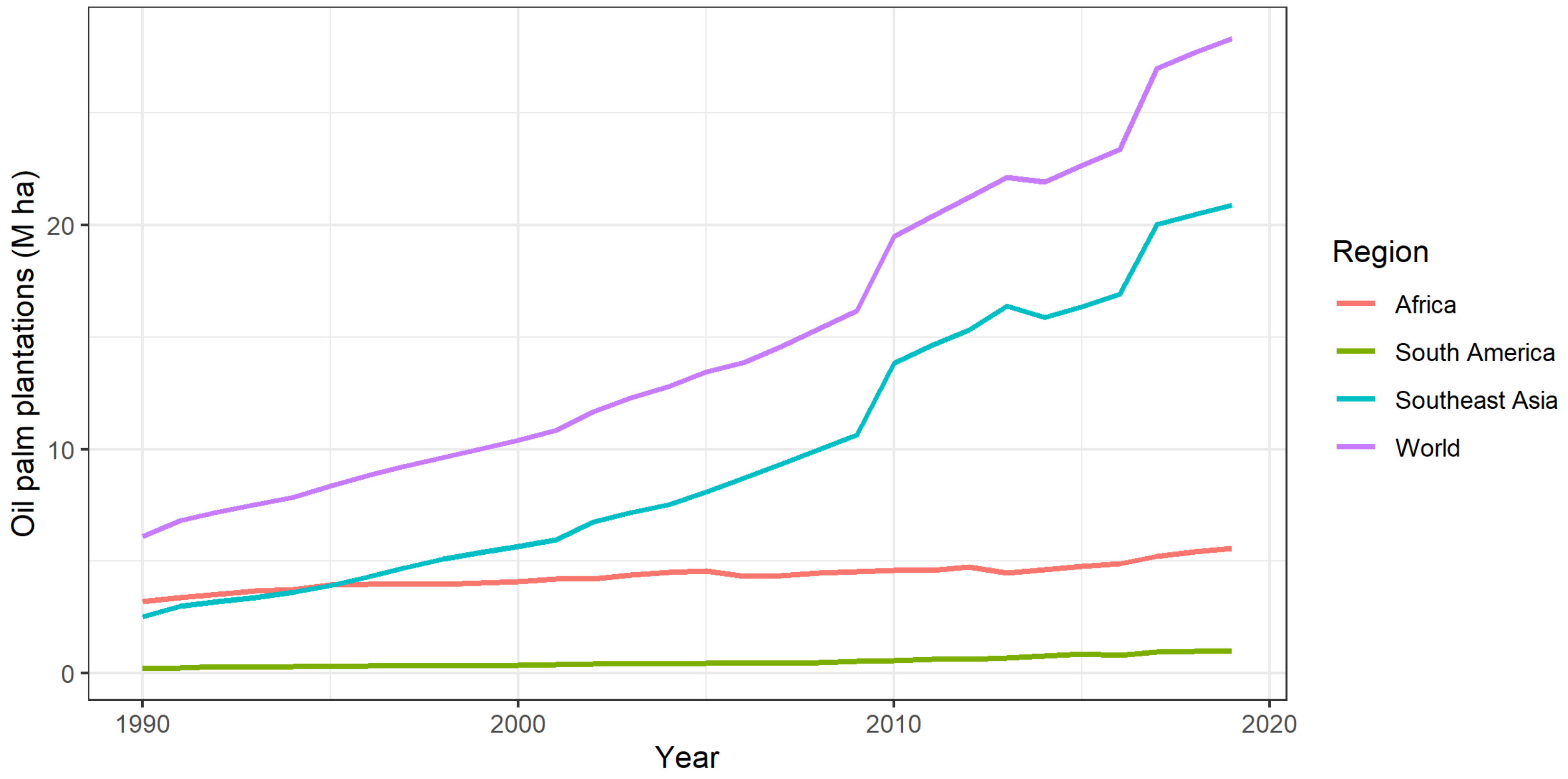

Figure S1 – Evolution of oil palm plantations in M ha per world region between 1990 – 2019. Related to Figure 1.

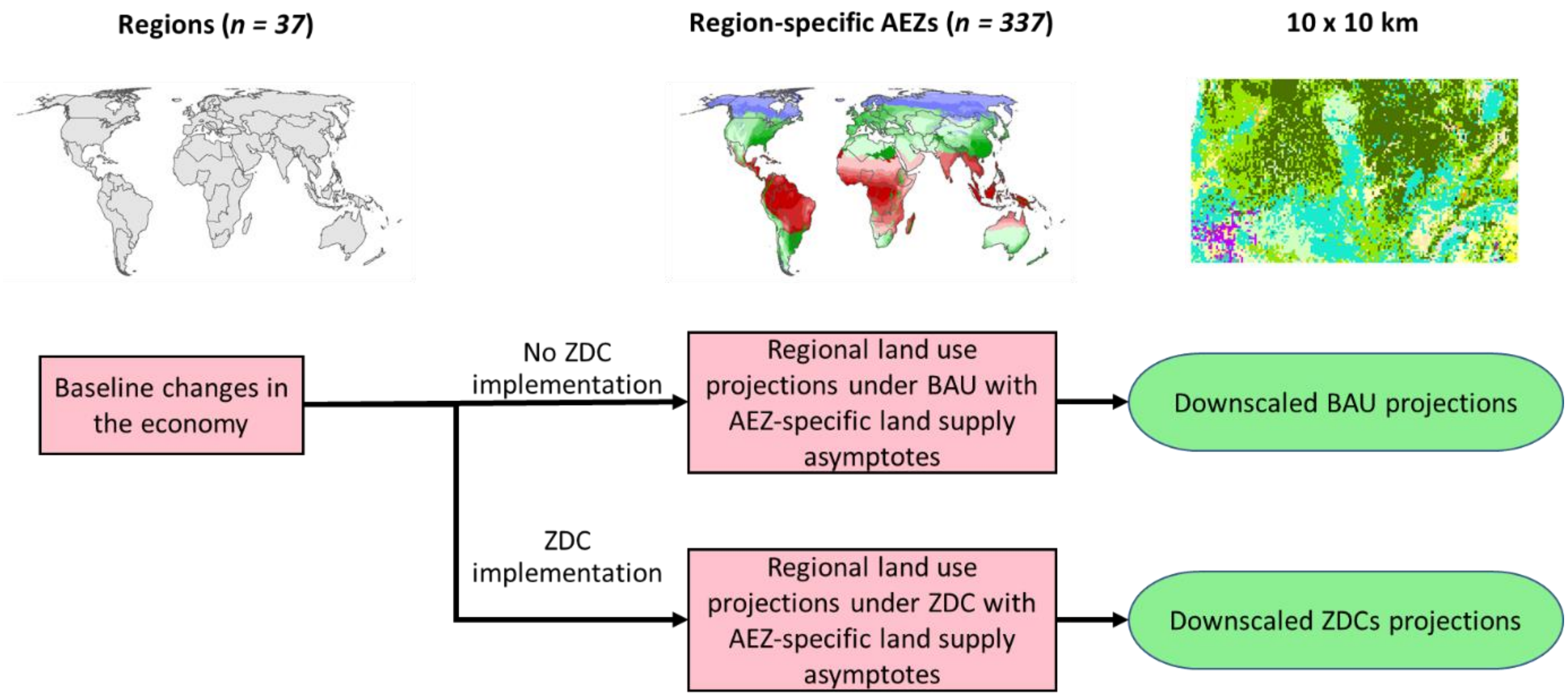

Figure S2 – Flowchart of the methodology. Maps above the flowchart indicate the spatial resolution of each step in the modelling process. Downscaled land use projections are only made for oil palm-producing regions. AEZs denotes Ago-Ecological Zones. BAU denotes Business-As-Usual. ZDCs denotes zero-deforestation commitments. Related to Figure 1.

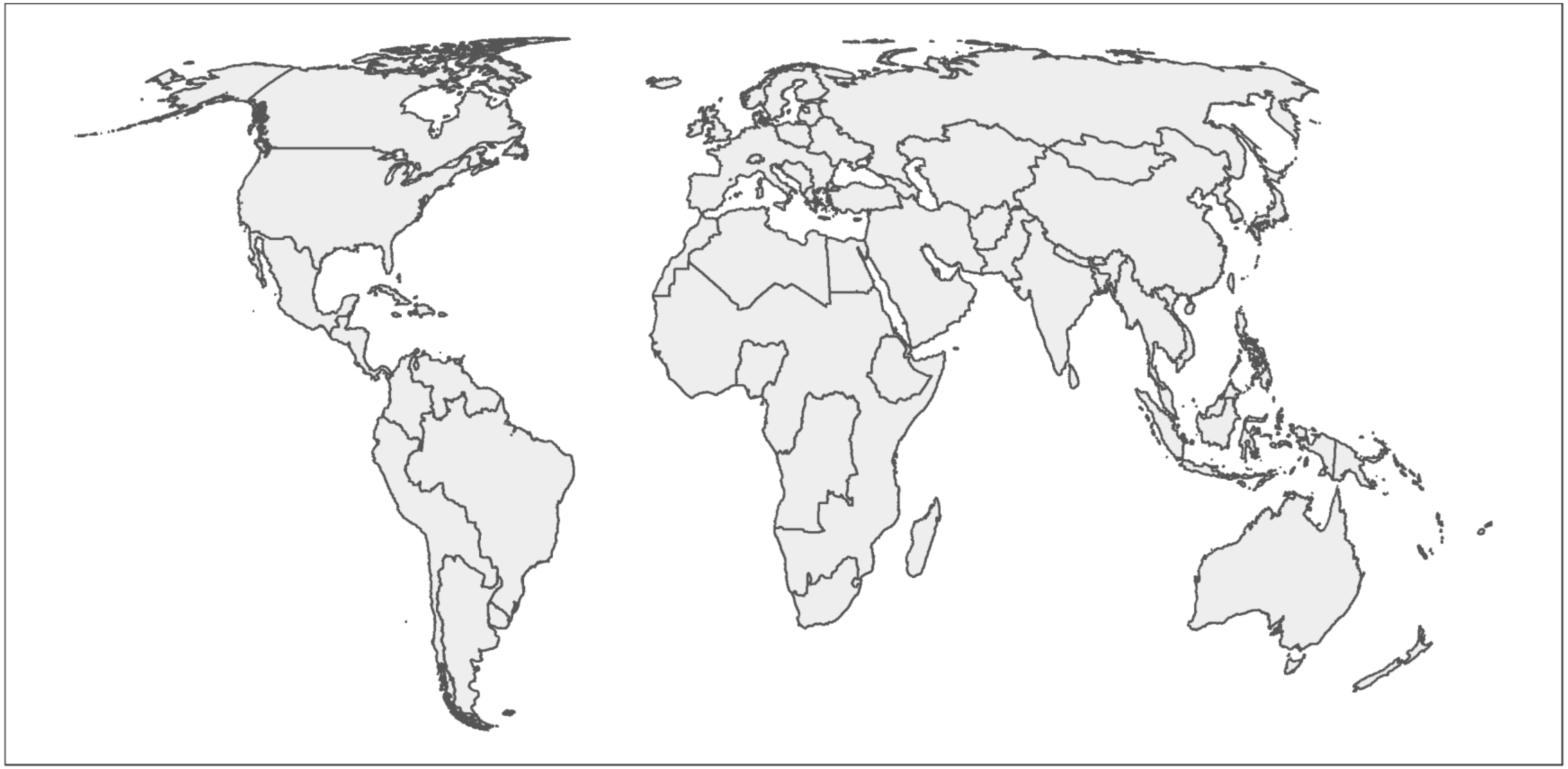

Figure S3 – Spatial overview of the 37 different regions in the GTAP-AEZ database. Related to Figure 1.

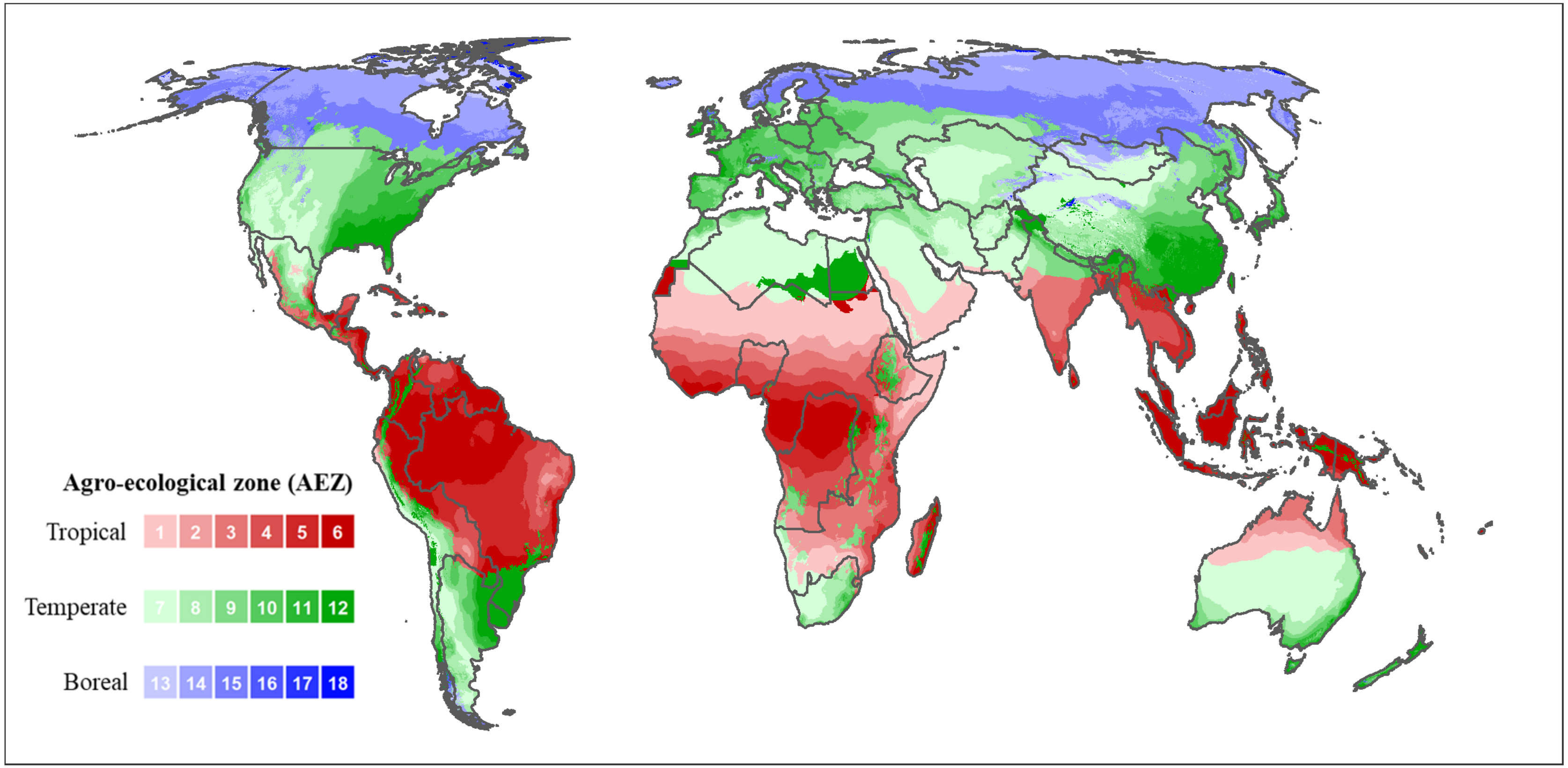

Figure S4 – Spatial overview of the 18 agro-ecological zones (AEZ) in the GTAP-AEZ database. AEZs are overlayed on the 37 regions in the GTAP-AEZ database. Related to Figure 1.

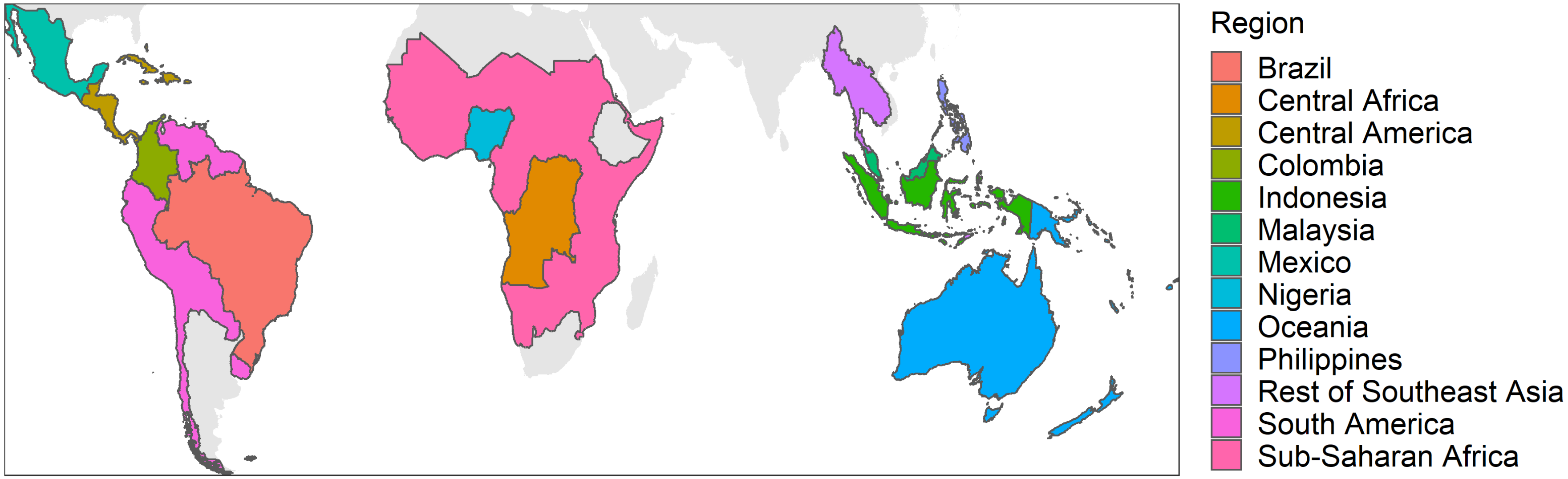

Figure S5 – Spatial overview of the 13 oil palm-producing regions in the GTAP-AEZ database. Related to Figure 1.

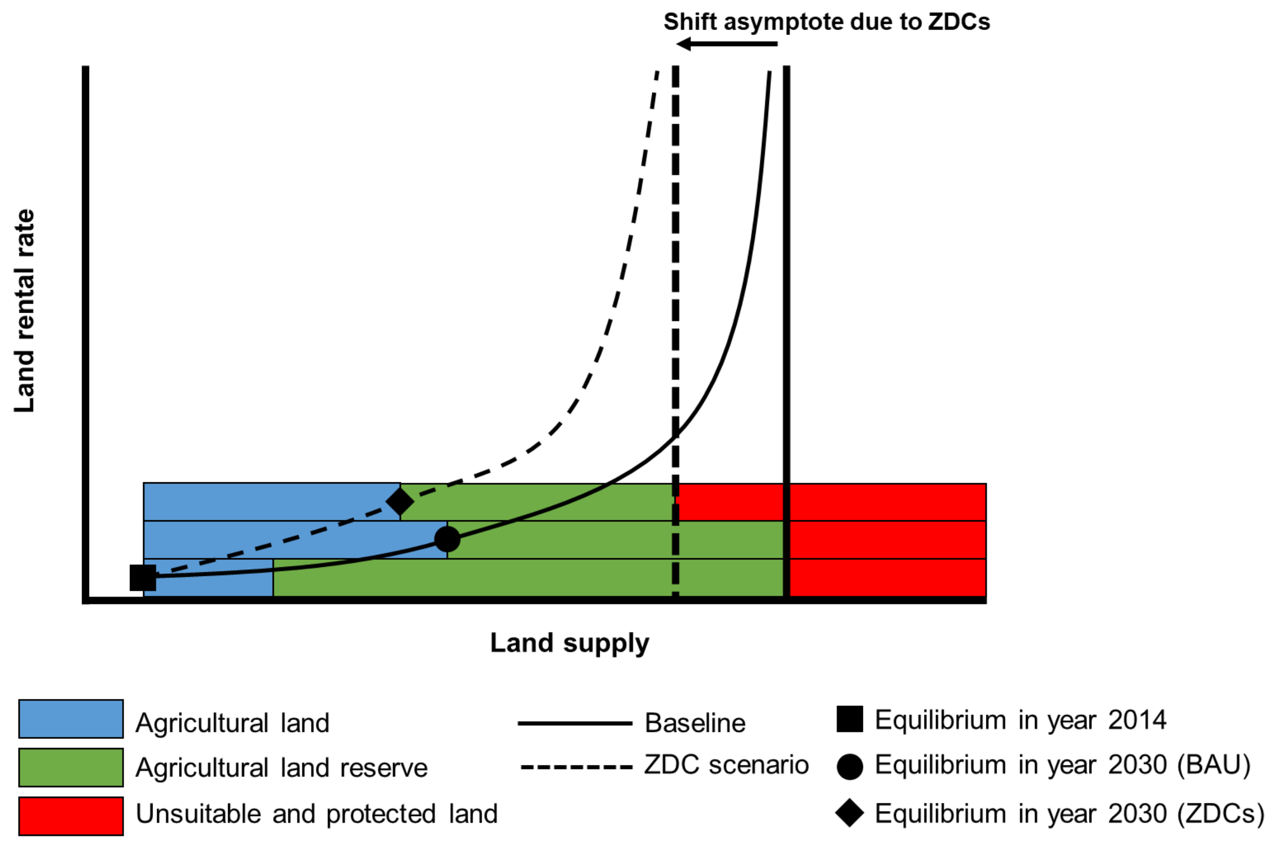

Figure S6 – Stylized graph – adapted from Overmars et al (2014) – showing the relationship between land rental rates and the supply of land under two scenarios: a baseline scenario assuming no compliance with zero-deforestation commitments (ZDCs) and an alternative scenario assuming full implementation of ZDCs. Land availability is constrained by the land supply asymptote: all areas beyond the asymptote are assumed to be unavailable for agricultural production. Implementation of ZDCs implies a leftward shift of the land supply asymptote as it involves an increase in the area that is protected from agricultural encroachment. Related to Figure 1.

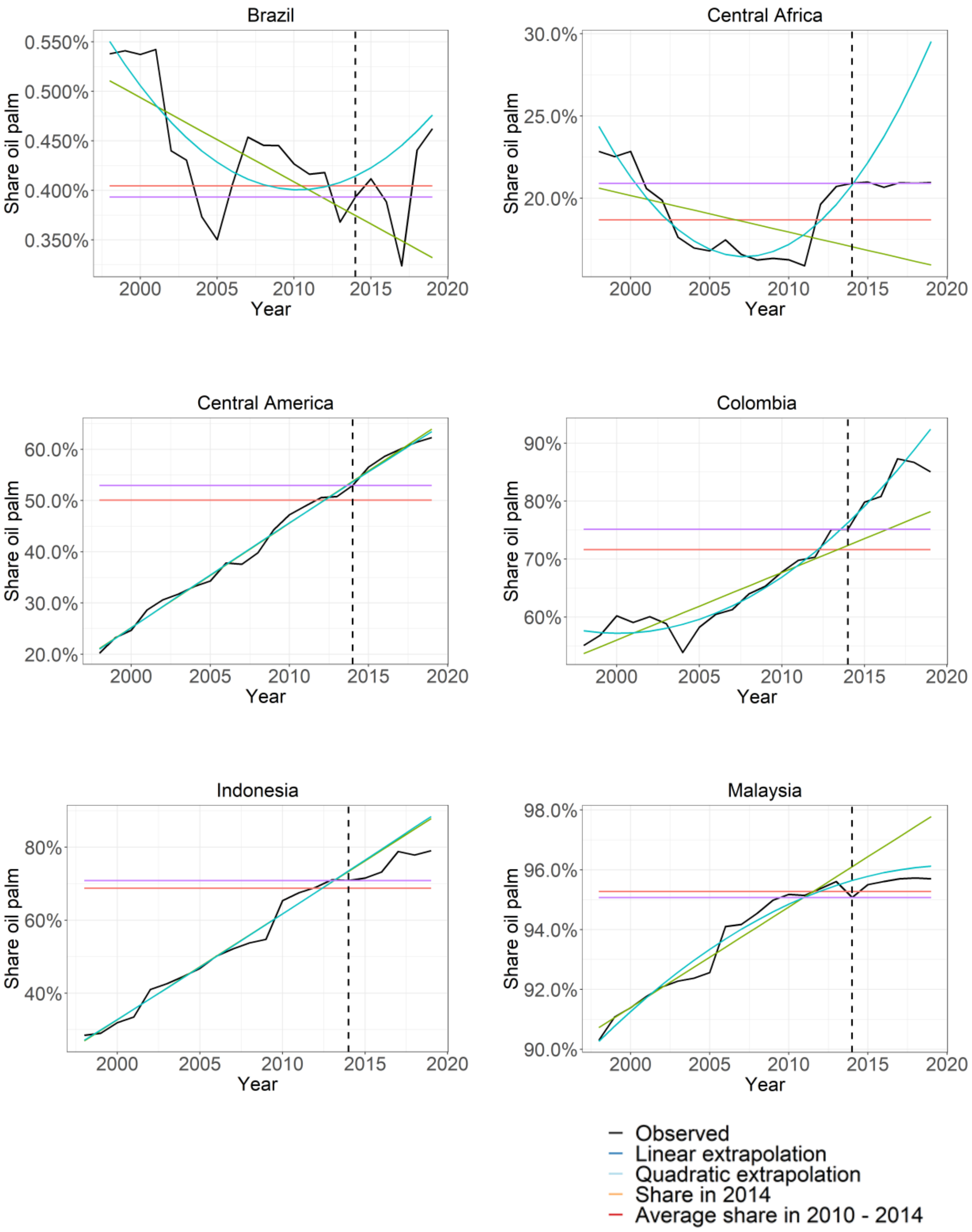

Figure S7a –Evolution of share of oil palm (%) relative to other oil crops for each oil palm producing region in the GTAP-AEZ database. To project the share up until 2030, 4 different approaches are considered and evaluated in terms of their Root Mean Square Error against the period 2015 – 2019. A spatial overview of the different regions is presented in Figure S5. Related to Figure 1.

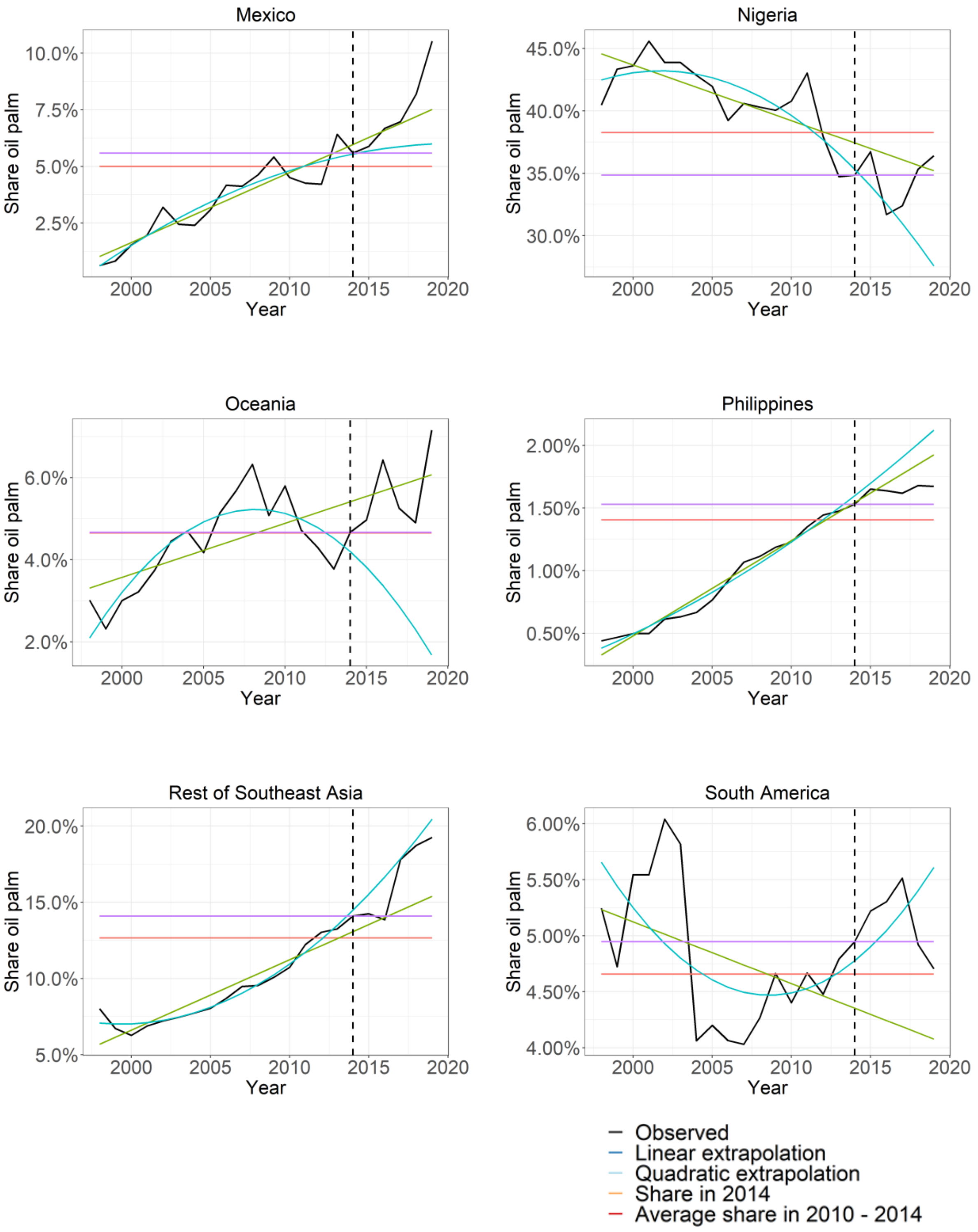

Figure S7b –Evolution of share of oil palm (%) relative to other oil crops for each oil palm producing region in the GTAP-AEZ database. To project the share up until 2030, 4 different approaches are considered and evaluated in terms of their Root Mean Square Error against the period 2015 – 2019. A spatial overview of the different regions is presented in Figure S5. Related to Figure 1.

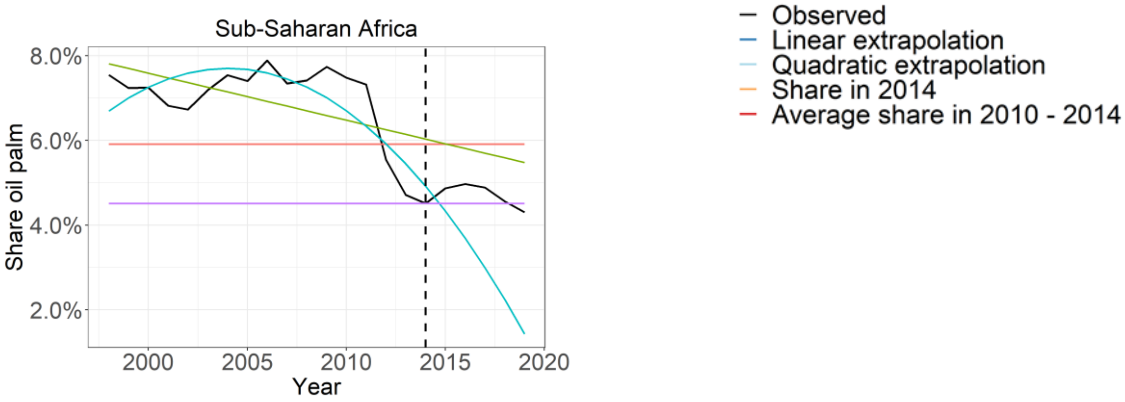

Figure S7c –Evolution of share of oil palm (%) relative to other oil crops for each oil palm producing region in the GTAP-AEZ database. To project the share up until 2030, 4 different approaches are considered and evaluated in terms of their Root Mean Square Error against the period 2015 – 2019. A spatial overview of the different regions is presented in Figure S5. Related to Figure 1.

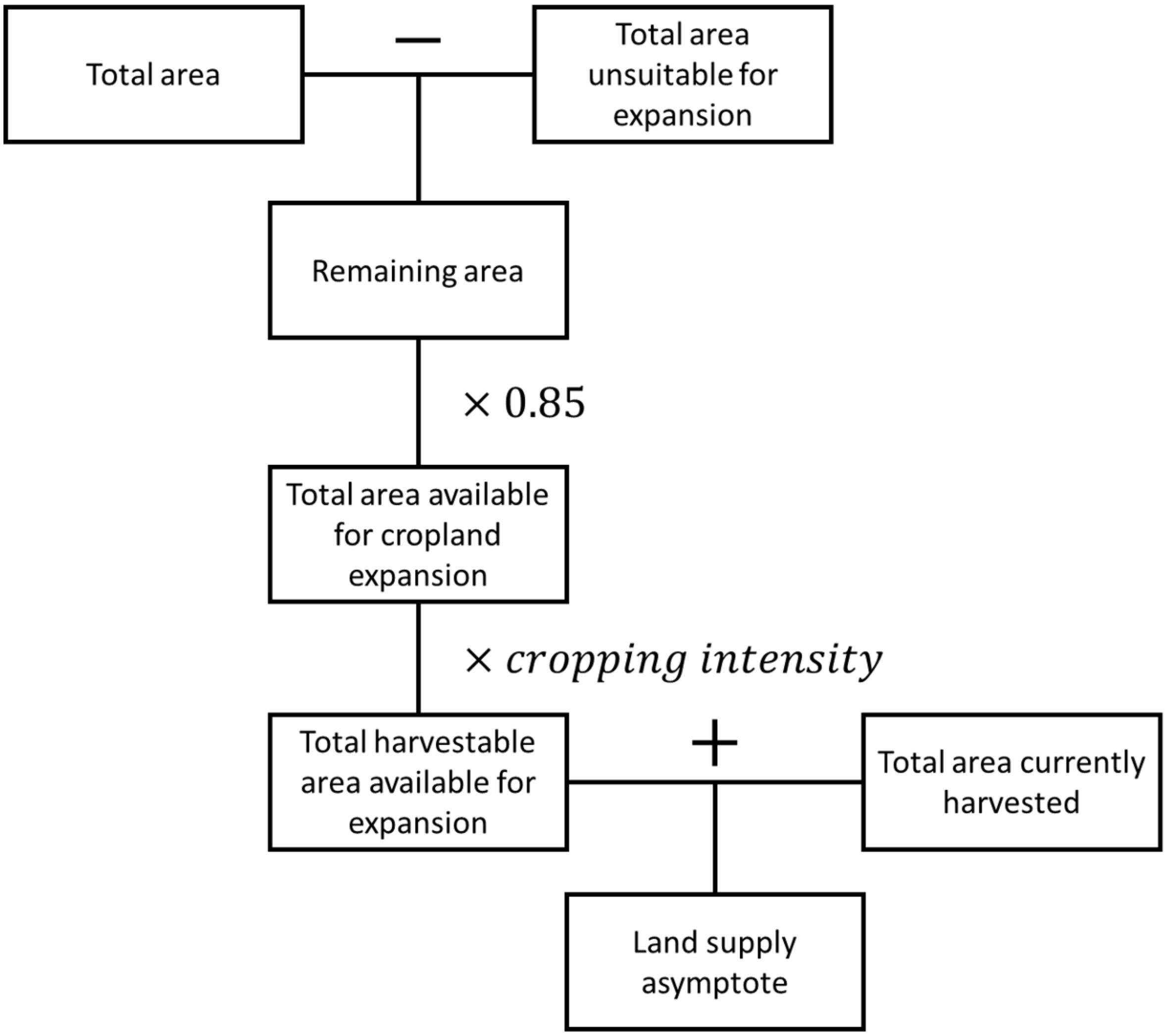

Figure S8 – Flowchart of the methodology to construct new land supply asymptotes within each region-specific agro-ecological zone. Related to Figure 1.

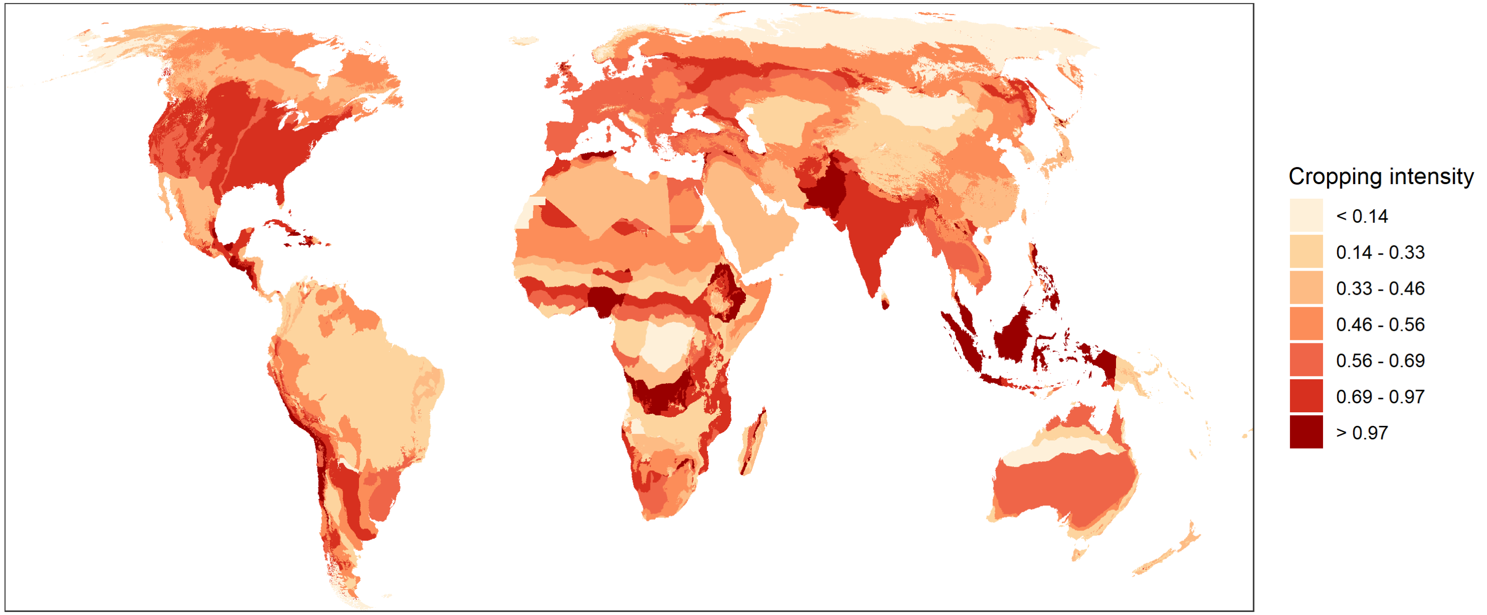

Figure S9 – Spatial overview of the estimated multiple cropping intensities in 2014 within each region-specific agro-ecological zone. Related to Figure 1.

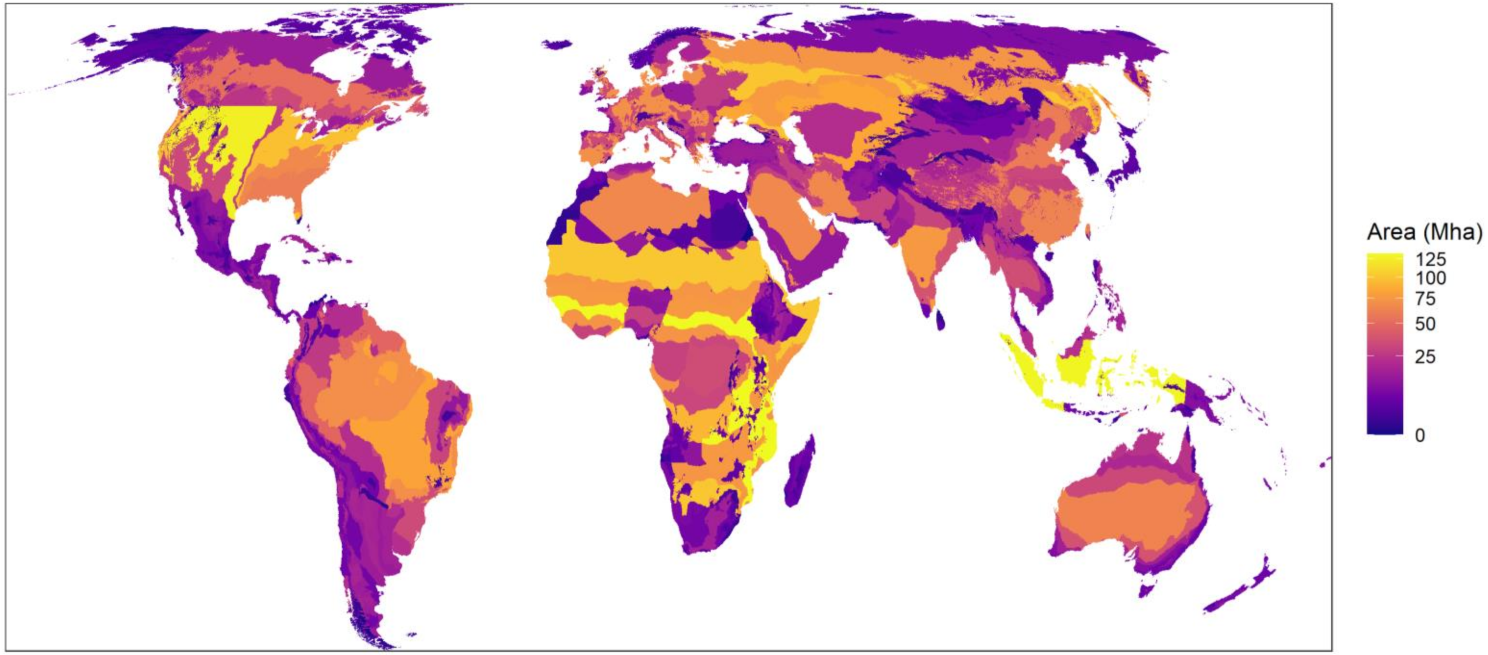

Figure S10a – Estimated land supply asymptotes (total area available for agriculture) as of 2014 assuming no implementation of zero-deforestation commitments. Related to Figure 1.

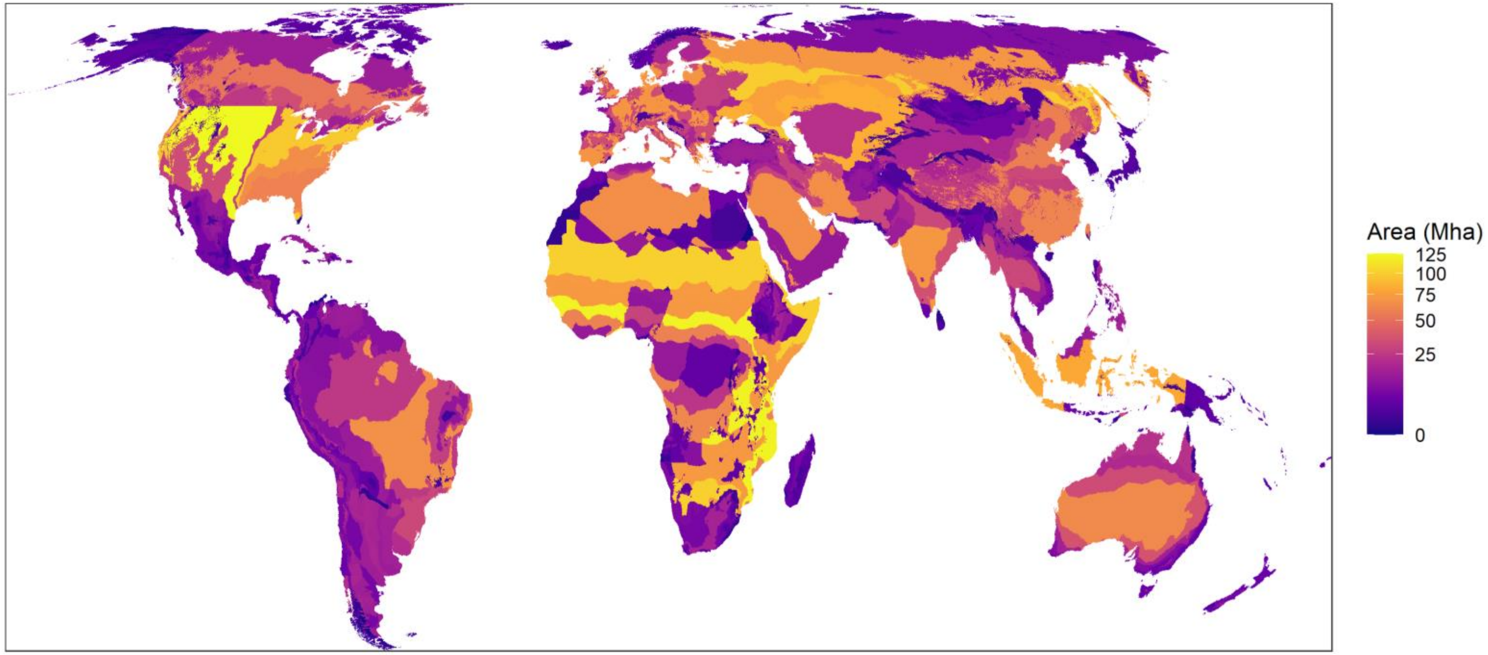

Figure S10b – Estimated land supply asymptotes (total area available for agriculture) as of 2014 assuming full implementation of zero-deforestation commitments. Related to Figure 1.

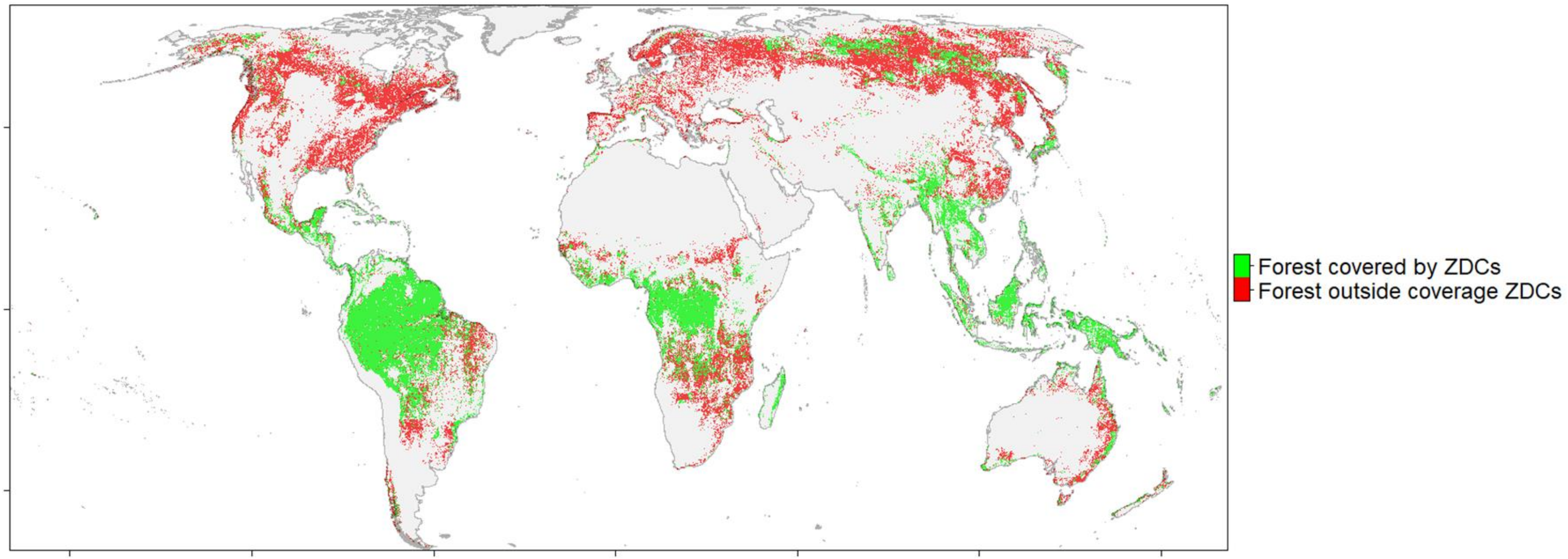

Figure S11 – Estimated coverage of zero-deforestation commitments based on the likely distribution of high conservation value forests and high carbon stock forests. Data obtained from Leijten et al (2020). Related to Figure 1.

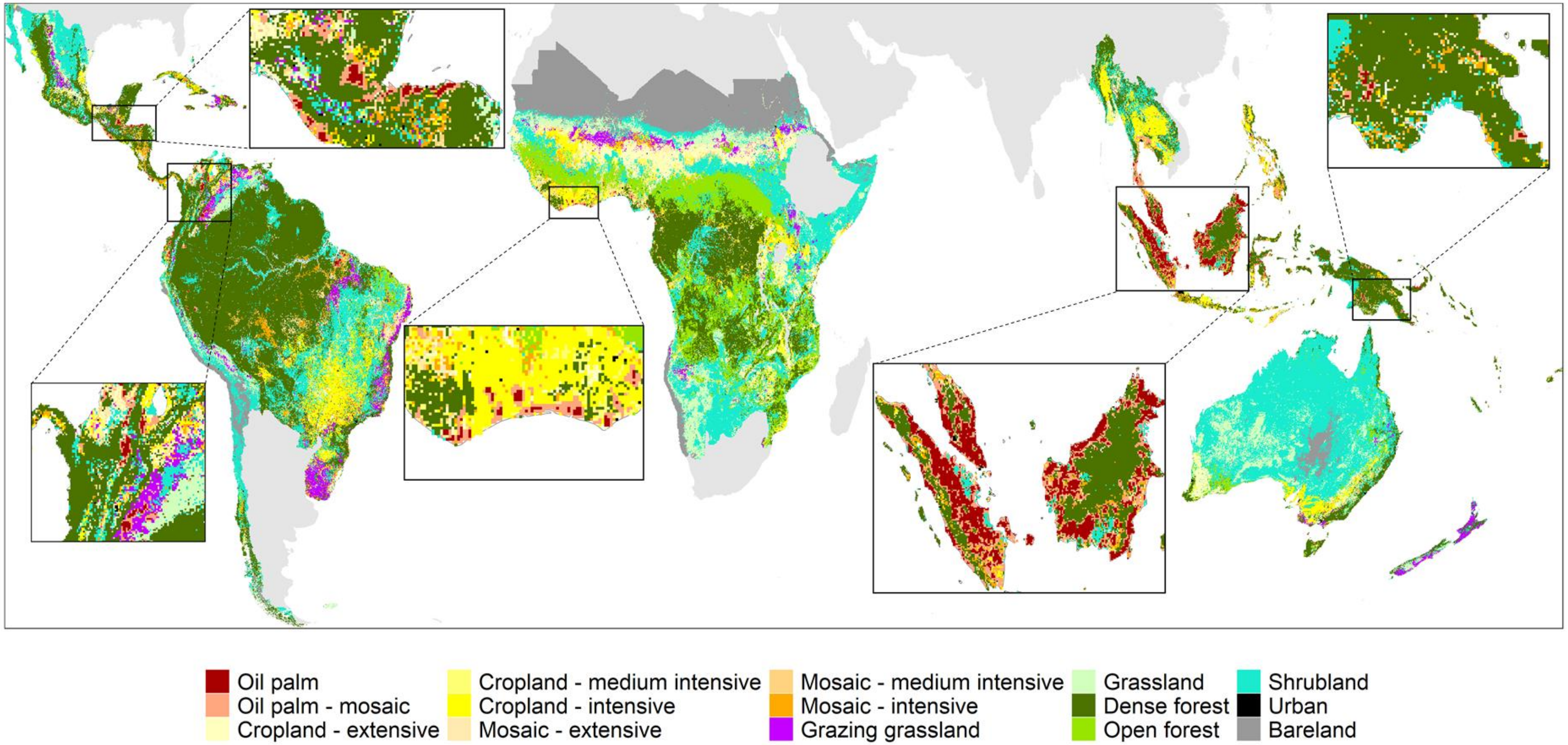

Figure S12 – Initial land use map used in the CLUMondo simulations at 10 x 10 km resolution. Zoom maps zero in on major oil palm-producing regions. Non- oil palm-producing regions are greyed out. Reference year is 2014. Related to Figure 1.

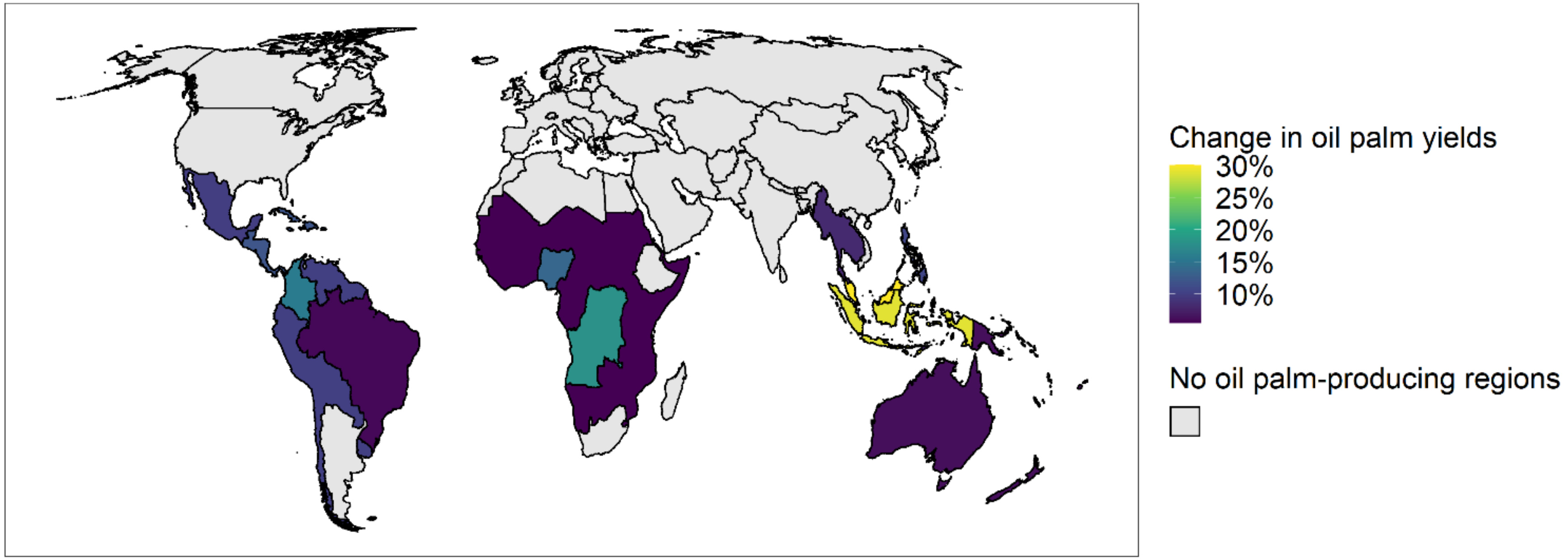

Figure S13 – Spatial overview of the impact of zero-deforestation commitments on oil palm yields in 2030 (relative to the BAU scenario). Related to Figure 2.

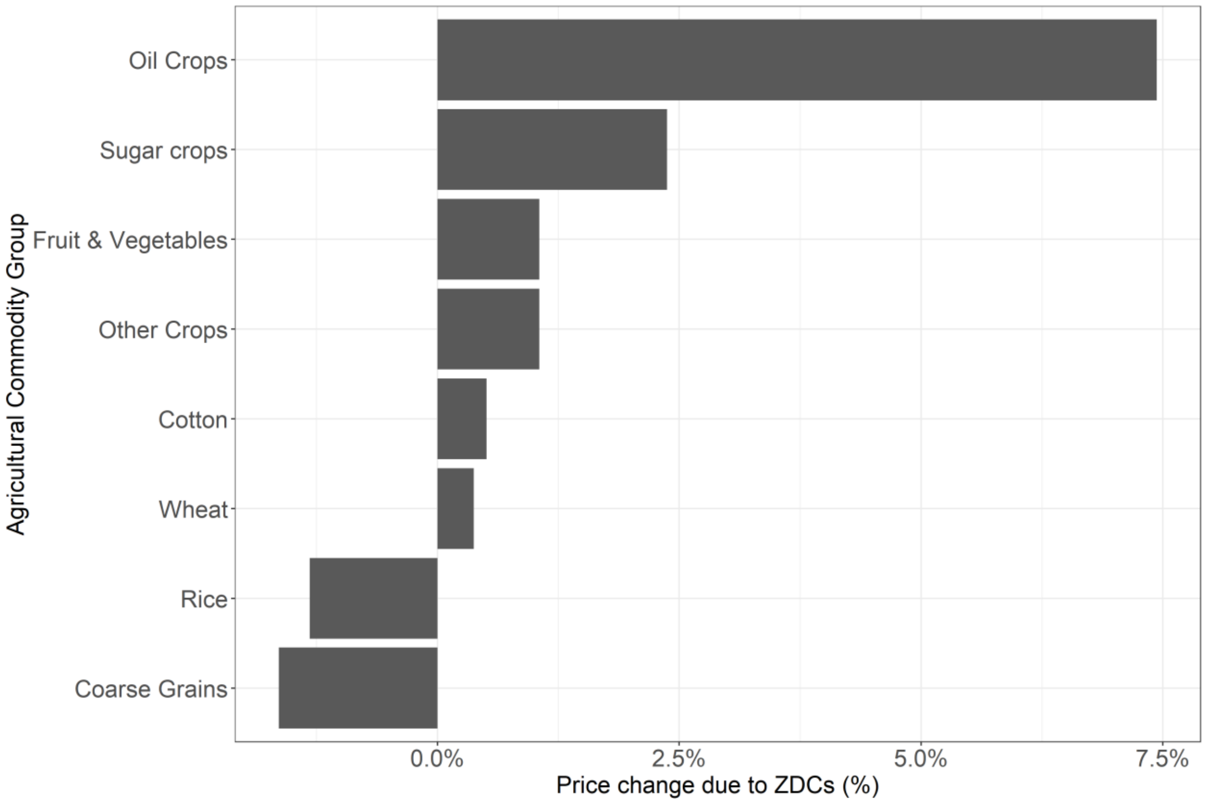

Figure S14 – Projected changes in global commodity prices by agricultural commodity group due to zero-deforestation commitments (ZDCs). Related to Figure 2.

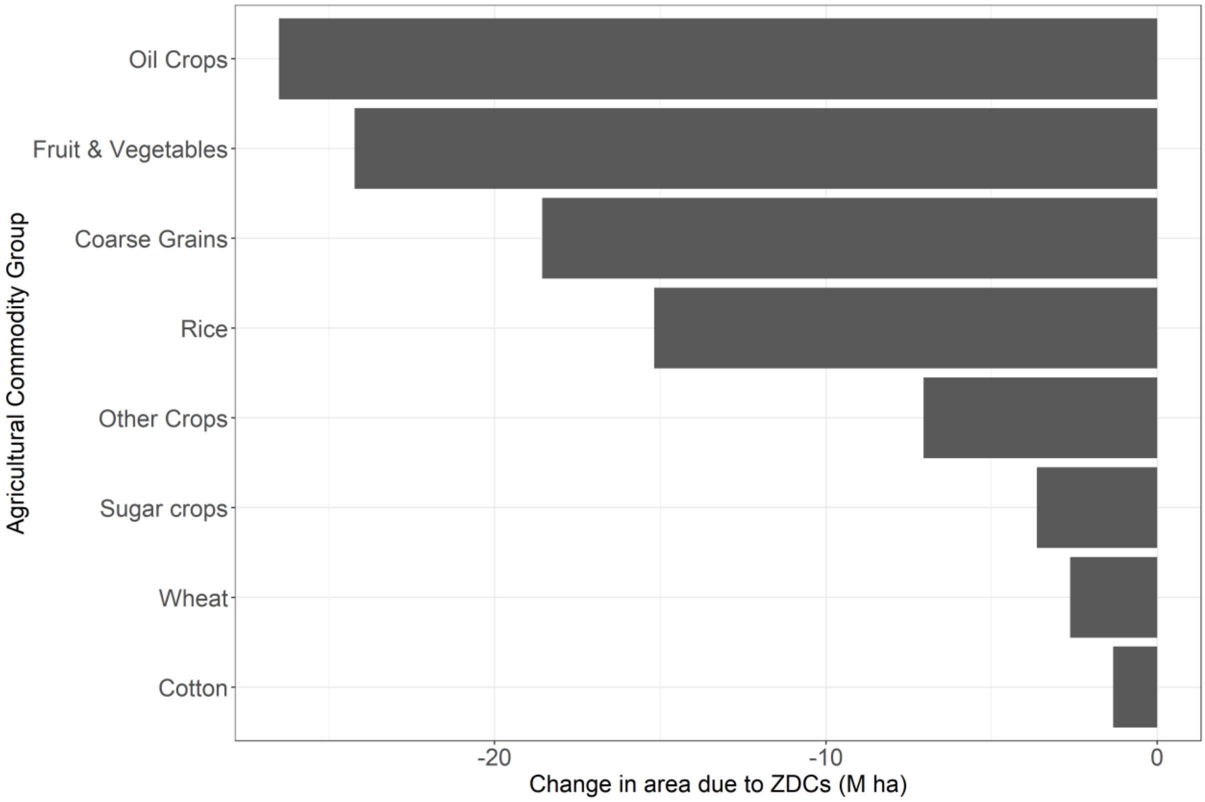

Figure S15 – Projected changes in area by agricultural commodity group within the oil palm-producing world due to zero-deforestation commitments (ZDCs). Related to Figure 2.

Appendix B – Table

Indicator	Source	Original resolution	Data processing	Classification rule
Agricultural suitability for the 16 most important food and energy crops based on climatic, soil and topographic conditions	Zabel et al (2014)	30 arc seconds (approximately 1 x 1km at the equator)	Resampled to a 1000 x 1000 m resolution using the nearest neighbour method	All grid cells classified as unsuitable by [1] were assumed to be unavailable for expansion
Existing cropland	ESA-CCI – Defourny et al (2017); reference year: 2014	10 arc seconds (approximately 300 x 300 m at the equator)	Resampled to a 1000 x 1000 m resolution using the majority resampling method	All areas classified as "Cropland, rainfed", "Cropland, irrigated or post-flooding" were assumed to be unavailable for expansion. For the two mosaic classes, ("Mosaic cropland (>50 %) / natural vegetation (< 50%)" and "Mosaic natural vegetation (>50 %) / cropland (< 50%)"), a cropland fraction of 58 and 38% was assumed, respectively
Legally protected areas	UNEP-WCMC & IUCN (2018)	N.A. (shapefile)	Rasterized to a 1000 x 1000 m grid	All protected areas were assumed to be unavailable for expansion
Rough terrains (steep slopes)	Lloyd (2016)	100 x 100 m	Resampled to a 1000 x 1000 m resolution using the majority resampling method	Region-specific slope threshold based on the top 5% slope values within existing cropland areas. A minimum threshold of 10 degrees was imposed.
Urban areas	ESA-CCI – Defourny et al (2017); reference year: 2014	10 arc seconds (approximately 300 x 300 m at the equator)	Resampled to a 1000 x 1000 m resolution using the majority resampling method	All areas classified as "Urban areas" were assumed to be unavailable for expansion

Table S1 – Criteria used to identify areas unsuitable for cropland expansion. Related to Figure 1.

Value	Label	Reclassified into
0	No Data	No Data
10	Cropland, rainfed	Cropland
11	Herbaceous cover	
12	Tree or shrub cover	
20	Cropland, irrigated or post-flooding	
30	Mosaic cropland (>50%) / natural vegetation (tree, shrub, herbaceous cover) (<50%)	Mosaic cropland
40	Mosaic natural vegetation (tree, shrub, herbaceous cover) (>50%) / cropland (<50%)	
50	Tree cover, broadleaved, evergreen, closed to open (>15%)	Dense forest
60	Tree cover, broadleaved, deciduous, closed to open (>15%)	
61	Tree cover, broadleaved, deciduous, closed (>40%)	
62	Tree cover, broadleaved, deciduous, open (15-40%)	Open forest
70	Tree cover, needleleaved, evergreen, closed to open (>15%)	Dense forest
71	Tree cover, needleleaved, evergreen, closed (>40%)	
72	Tree cover, needleleaved, evergreen, open (15-40%)	Open forest
80	Tree cover, needleleaved, deciduous, closed to open (>15%)	Dense forest
81	Tree cover, needleleaved, deciduous, closed (>40%)	
82	Tree cover, needleleaved, deciduous, open (15-40%)	Open forest
90	Tree cover, mixed leaf type (broadleaved and needleleaved)	Dense forest
100	Mosaic tree and shrub (>50%) / herbaceous cover (<50%)	Shrubland; herbaceous cover; lichens and mosses; sparse vegetation; flooded tree cover.
110	Mosaic herbaceous cover (>50%) / tree and shrub (<50%)	
120	Shrubland	
121	Evergreen shrubland	
122	Deciduous shrubland	

130	Grassland	Grassland
140	Lichens and mosses	Shrubland; herbaceous cover; lichens and mosses; sparse vegetation; flooded tree cover.
150	Sparse vegetation (tree, shrub, herbaceous cover) (<15%)	
151	Sparse tree (<15%)	
152	Sparse shrub (<15%)	
153	Sparse herbaceous cover (<15%)	
160	Tree cover, flooded, fresh or brakish water	
170	Tree cover, flooded, saline water	
180	Shrub or herbaceous cover, flooded, fresh/saline/brakish water	
190	Urban areas	Urban areas
200	Bare areas	Bare areas
201	Consolidated bare areas	
202	Unconsolidated bare areas	
210	Water bodies	No Data
220	Permanent snow and ice	Bare areas

Table S2 – Overview of the ESA CCI-LC 2014 land cover classes. The last column shows the different classifications that were used to construct the land systems map for the year 2014. Related to Figure 1.

Predictor variable	Source	Predictor variable	Source
Actual evapotranspiration	Abatzoglou et al (2018)	Precipitation	Abatzoglou et al 2018
Altitude	Amatulli et al (2018)	Presence indigenous community	Garnett et al (2018)
Aspect cosine	Amatulli et al (2018)	Proportion population aged between 15 - 64	CIESIN (2018)
Aspect sine	Amatulli et al (2018)	Proportion population female	CIESIN (2018)
Cation exchange capacity	De Sousa et al (2020)	Reference evapotranspiration	Abatzoglou et al 2018
Clay	De Sousa et al (2020)	Runoff	Abatzoglou et al 2018
Climate water deficit	Abatzoglou et al 2018	Sand	De Sousa et al (2020)
Coarse fragments in the soil	De Sousa et al (2020)	Shannon index geomorphological landforms	Amatulli (2018)
Downward surface shortwave radiation	Abatzoglou et al 2018	Silt	De Sousa et al (2020)
Gross Domestic Product per capita	Kummu et al (2018)	Slope	Amatulli (2018)
Human Development Index per capita	Kummu et al 2018	Snow water equivalent	Abatzoglou et al 2018
Human footprint index	Venter et al (2018)	Soil bulk density	De Sousa et al (2020)
Irrigation	Siebert et al (2015)	Soil moisture	Abatzoglou et al 2018
Maximum temperature	Abatzoglou et al 2018	Soil organic carbon	De Sousa et al (2020)
Minimum temperature	Abatzoglou et al 2018	Soil pH	De Sousa et al (2020)
Nitrogen	de Sousa et al (2020)	Suitability plantation forest	Schulze et al (2019)
NPP of potential vegetation	Haberl et al (2007)	Travel time to nearest city	Weiss et al (2018)
Organic carbon density	De Sousa et al (2020)	Travel time to nearest palm oil mill	Leijten et al (2021)
Organic carbon stock	De Sousa et al (2020)	Travel time to nearest port	Weiss et al (2018)
Palmer Drought Severity Index	Abatzoglou et al 2018	Vapor pressure	Abatzoglou et al 2018
Population	Gao (2017)	Vapor pressure deficit	Abatzoglou et al 2018
Population - rural	Gao (2017)	Wind-speed	Abatzoglou et al 2018

Table S3 – All 44 predictors variables used in the logistic regression analysis. Climatological variables were averaged over the period 2010 – 2019. All raster data were resampled to a 10 x 10 km grid using the bilinear resampling method. Related to Figure 1

	Cropland - extensive	Mosaic - extensive	Dense forest	Open forest	Shrubland and herbaceous	Grassland	Urban	Bareland	Oil palm - mosaic	Oil palm	Cropland - medium intensive	Mosaic - medium intensive	Cropland - intensive	Mosaic - intensive	Grazing grassland
**Cropland - extensive**	1	1	0	0	1	1	1	0	0	0	1	1	1	1	1
**Mosaic - extensive**	1	1	0	0	1	1	1	0	103	103	1	1	1	1	1
**Dense forest**	1	1	1	1	0	0	1	0	103	103	1	1	1	1	1
**Open forest**	1	1	130	1	0	0	1	0	103	103	1	1	1	1	1
**Shrubland and herbaceous**	1	1	0	120	1	1	1	0	103	103	1	1	1	1	1
**Grassland**	1	1	0	0	1	1	1	0	103	0	1	1	1	1	1
**Urban**	0	0	0	0	0	0	1	0	0	0	0	0	0	0	0
**Bareland**	0	0	0	0	0	0	0	1	0	0	0	0	0	0	0
**Oil palm - mosaic**	1	1	0	0	1	1	1	0	1	103	1	1	0	0	1
**Oil palm**	1	1	0	1	1	1	0	0	1	1	1	1	0	0	1
**Cropland - medium intensive**	1	1	0	0	1	1	1	0	0	0	1	1	1	1	1
**Mosaic - medium intensive**	1	1	0	0	1	1	1	0	103	103	1	1	1	1	1
**Cropland - intensive**	1	1	0	0	1	1	1	0	0	0	1	1	1	1	1
**Mosaic - intensive**	1	1	0	0	1	1	1	0	103	103	1	1	1	1	1
**Grazing grassland**	1	1	0	0	1	1	1	0	103	0	1	1	1	1	1

Table S4 – Land conversion matrix used in the CLUMondo simulations. A value of "1" indicates that the conversion is allowed and a value of "0" indicates it is not. Three-digit values starting with a "1" indicate conversions that are only allowed after a minimum number of years, with the last two numbers indicating the minimum number of years. For example, a value of "103" indicates that the conversion from "Dense forest" into "Oil palm" can only occur after a period of 3 years. Related to Figure 3.

	Cropland - extensive	Mosaic - extensive	Dense forest	Open forest	Shrubland and herbaceous	Grassland	Urban	Bareland	Oil palm - mosaic	Oil palm	Cropland - medium intensive	Mosaic - medium intensive	Cropland - intensive	Mosaic - intensive	Grazing grassland
**Angola**	0.1	0	0.1	0	0	0	1	1	0.6	1	0.15	0	0.25	0.1	0.2
**Australia**	0.1	0	0.1	0	0	0	1	1	0.6	1	0.15	0	0.25	0.1	0.2
**Brazil**	0.1	0	0.1	0	0	0	1	1	0.6	1	0.15	0	0.25	0.1	0.2
**Chile**	0.1	0	0.1	0	0	0	1	1	0.6	1	0.15	0	0.25	0.1	0.2
**Colombia**	0.1	0	0.1	N.A.	0	0	1	N.A.	0.6	1	0.15	0	0.25	0.1	0.2
**East Timor - R SE Asia**	0.1	0	0.1	N.A.	0	0	1	N.A.	0.6	1	0.15	0	0.25	0.1	0.2
**Indonesia**	0.1	0	0.1	N.A.	0	N.A.	1	N.A.	0.5	0.7	0.15	0	0.25	0.1	N.A.
**Jamaica**	0.1	0	0.1	N.A.	0	0	1	N.A.	0.6	1	0.15	0	0.25	0.1	0.2
**Kenya**	0.1	0	0.1	0	0	0	1	1	0.6	1	0.15	0	0.25	0.1	0.2
**Malaysia**	0.1	0	0.1	N.A.	0	N.A.	1	N.A.	0.5	0.7	N.A.	N.A.	N.A.	N.A.	N.A.
**Mexico**	0.1	0	0.1	N.A.	0	0	1	1	0.6	1	0.15	0	0.25	0.1	0.2
**Nigeria**	0.1	0	0.1	0	0	0	1	N.A.	0.6	1	0.15	0	0.25	0.1	0.2
**Philippines**	0.1	0	0.1	N.A.	0	N.A.	1	N.A.	0.6		0.15	0	0.25	0.1	N.A.
**Mosaic - intensive**	0.1	0	0.1	0	0	0	1	1	0.6	1	0.15	0	0.25	0.1	0.2
**Grazing grassland**	0.1	0	0.1	0	0	0	1	1	0.6	1	0.15	0	0.25	0.1	0.2

Table S5 – Conversion resistance elasticities ($Pres_{LS}$) for each land system used in the CLUMondo simulations. Related to Figure 3.

	Demand type				
	**Forestry (ha)**	**Oil palm (ha)**	**Other agricultural commodities (ha and metric tonnes)**	**Pastureland (ha)**	**Urban (ha)**
Cropland - extensive			x		
Cropland - intensive			x		
Cropland - medium intensive			x		
Dense forest	x				
Grassland				x	
Grazing grassland				x	
Mosaic - extensive	x		x		
Mosaic - intensive	x		x		
Mosaic - medium intensive	x		x		
Oil palm		x			
Oil palm - mosaic		x			
Open forest	x				
Shrubland and herbaceous					
Urban					x

Table S6 – Mapping of demand types to land systems used in CLUMondo. An “x” means that the demand type indicated in the column name is supplied by the land system indicated in the corresponding row. ‘Other agricultural commodities’ represents the residual of the output of all agricultural sectors in the GTAP database after subtracting oil palm output. Related to Figure 3.

**Land system**	Neighbourhood weight factor
Cropland - extensive	0.2
Mosaic - extensive	0.1
Dense forest	0.5
Open forest	0.3
Shrubland and herbaceous	0.4
Grassland	0.1
Urban	1
Oil palm - mosaic	0.8
Oil palm	1
Cropland - medium intensive	0.2
Mosaic - medium intensive	0.1
Cropland - intensive	0.2
Mosaic - intensive	0.1
Grazing grassland	0.1

Table S7 – List of neighbourhood weight factors by land system. Related to Figure 3.

References

1. Zabel, F., Putzenlechner, B., and Mauser, W. (2014). Global agricultural land resources - A high resolution suitability evaluation and its perspectives until 2100 under climate change conditions. PLoS One *9*. 10.1371/journal.pone.0107522.

2. Defourny, P., Bontemps, S., Lamarche, C., Brockmann, C., Boettcher, M., Wevers, J., Kirches, G., Santoro, M., and ESA (2017). Land Cover CCI Product User Guide - Version 2.0. ESA.

3. UNEP-WCMC and IUCN (2018). Protected Planet: The World Database on Protected Areas (WDPA)/The Global Database on Protected Areas Management Effectiveness (GD-PAME)] [On-line], [23/11/2018]. https://www.protectedplanet.net/.

4. Lloyd, C.T. (2016). WorldPop Archive global gridded spatial datasets. Version Alpha 0.9. 100m base topography (tiled). Harvard Dataverse, V1 *https://do*. 10.1038/sdata.2017.1.

5. Abatzoglou, J.T., Dobrowski, S.Z., Parks, S.A., and Hegewisch, K.C. (2018). TerraClimate, a high-resolution global dataset of monthly climate and climatic water balance from 1958-2015. Sci Data. 10.1038/sdata.2017.191.

6. Amatulli, G., Domisch, S., Tuanmu, M.N., Parmentier, B., Ranipeta, A., Malczyk, J., and Jetz, W. (2018). Data Descriptor: A suite of global, cross-scale topographic variables for environmental and biodiversity modeling. Sci Data *5*. 10.1038/sdata.2018.40.

7. Garnett, S.T., Burgess, N.D., Fa, J.E., Fernández-Llamazares, Á., Molnár, Z., Robinson, C.J., Watson, J.E.M., Zander, K.K., Austin, B., Brondizio, E.S., et al. (2018). A spatial overview of the global importance of Indigenous lands for conservation. Nat Sustain *1*, 369–374. 10.1038/s41893-018-0100-6.

8. CIESIN (2018). Gridded Population of the World, Version 4 (GPWv4): Basic Characteristics, Revision 11. Palisades, NY: NASA Socioeconomic Data and Applications Center (SEDAC). Columbia University Center for International Earth Science Information Network (CIESIN) - Columbia University.

9. de Sousa, L., Poggio, L., Batjes, N., Heuvelink, G., Kempen, B., Riberio, E., and Rossiter, D. (2020). SoilGrids 2.0: producing quality-assessed soil information for the globe. SOIL Discussions. 10.5194/soil-2020-65.

10. Kummu, M., Taka, M., and Guillaume, J.H.A. (2018). Gridded global datasets for Gross Domestic Product and Human Development Index over 1990–2015. Sci Data *5*, 180004. 10.1038/sdata.2018.4.

11. Venter, O., Sanderson, E.W., Magrach, A., Allan, J.R., Beher, J., Jones, K.R., Possingham, H.P., Laurance, W.F., Wood, P., Fekete, B.M., et al. (2018). Last of the Wild Project, Version 3 (LWP-3): 2009 Human Footprint, 2018 Release. NASA Socioeconomic Data and Applications Center *3*.

12. Siebert, S., Kummu, M., Porkka, M., Döll, P., Ramankutty, N., and Scanlon, B.R. (2015). A global data set of the extent of irrigated land from 1900 to 2005. Hydrol Earth Syst Sci *19*. 10.5194/hess-19-1521-2015.

13. Schulze, K., Malek, Ž., and Verburg, P.H. (2019). Towards better mapping of forest management patterns: A global allocation approach. For Ecol Manage *432*, 776–785. 10.1016/j.foreco.2018.10.001.

14. Haberl, H., Erb, K.H., Krausmann, F., Gaube, V., Bondeau, A., Plutzar, C., Gingrich, S., Lucht, W., and Fischer-Kowalski, M. (2007). Quantifying and mapping the human appropriation of net primary production in earth's terrestrial ecosystems. Proc Natl Acad Sci U S A *104*. 10.1073/pnas.0704243104.

15. Weiss, D.J., Nelson, A., Gibson, H.S., Temperley, W., Peedell, S., Lieber, A., Hancher, M., Poyart, E., Belchior, S., Fullman, N., et al. (2018). A global map of travel time to cities to assess inequalities in accessibility in 2015. Nature *553*, 333–336. 10.1038/nature25181.

16. Leijten, F., Sim, S., King, H., and Verburg, P.H. (2021). Local deforestation spillovers induced by forest moratoria: Evidence from Indonesia. Land use policy *109*, 105690. 10.1016/j.landusepol.2021.105690.

17. Gao, J. (2017). Downscaling Global Spatial Population Projections from 1/8-degree to 1-km Grid Cells. NCAR Technical Note.
